# Triglyceride cycling enables modification of stored fatty acids

**DOI:** 10.1038/s42255-023-00769-z

**Published:** 2023-04-03

**Authors:** Klaus Wunderling, Jelena Zurkovic, Fabian Zink, Lars Kuerschner, Christoph Thiele

**Affiliations:** LIMES Life and Medical Sciences Institute, University of Bonn, Bonn, Germany

**Keywords:** Fats, Biochemical assays, Fat metabolism

## Abstract

Triglyceride cycling is the process of continuous degradation and re-synthesis of triglyceride in cellular stores. We show in 3T3-L1 adipocytes that triglycerides are subject to rapid turnover and re-arrangement of fatty acids with an estimated half-life of 2–4 h. We develop a tracing technology that can simultaneously and quantitatively follow the metabolism of multiple fatty acids to study the triglyceride futile substrate cycle directly and with molecular species resolution. Our approach is based on alkyne fatty acid tracers and mass spectrometry. The triglyceride cycling is connected to modification of released fatty acids by elongation and desaturation. Through cycling and modification, saturated fatty acids are slowly converted to monounsaturated fatty acids, and linoleic acid to arachidonic acid. We conclude that triglyceride cycling renders stored fatty acids accessible for metabolic alteration. The overall process facilitates cellular adjustments to the stored fatty acid pool to meet changing needs of the cell.

## Abstract (web summary)

Wunderling et al. develop a fatty acid tracing technology based on alkyne-labelled fatty acids and directly demonstrate triglyceride cycling in 3T3-L1 adipocytes, which allows maintenance and modification of stored fatty acid pools.

## Main

Triglyceride/fatty acid (TG/FA) cycling is the process of partial or complete degradation of stored fat to release free FAs that subsequently are used to resynthesize a new molecule of TG. On the one hand, it can take place on the whole body level, where free FAs released from adipose tissue can be re-esterified to TGs in the liver, leading to the organismal redistribution of stored energy^1^. On the other hand, it also takes place intracellularly, as a typical ‘futile’ substrate cycle that consumes energy without a net synthesis of biological material^2^. Intracellular substrate cycling in general is a scientific challenge both conceptually and experimentally. On the conceptual side, the question is whether beneficial effects of substrate cycles might outbalance the energetic costs or whether substrate cycling is an unavoidable imperfection of complex networks. The early research on TG/FA cycling emphasized its regulatory role, in particular in adaptation to rapid changes in energy consumption^3,4^, while energetic costs of the cycle were considered to be small^5^. In recent years, however, a rising interest in the cycle has originated from the idea that it might substantially contribute to thermogenesis in adipose tissue^6,7^. A better understanding of thermogenic pathways might lead to new strategies for influencing energy expenditure, possibly useful in the fight against the global obesity pandemic^8,9^.

**Fig. 1:**
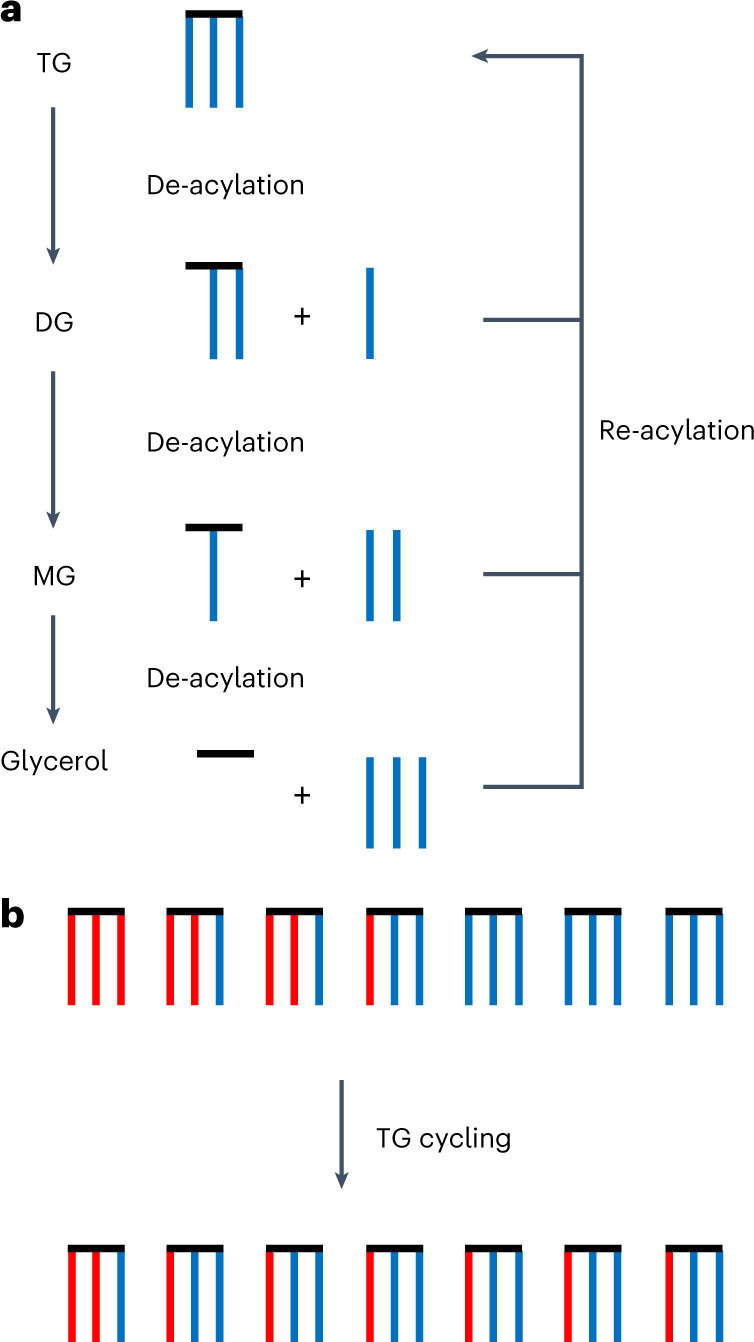
Tracing of TG cycling by dissipation of labelled FAs. TG cycling consists of permanent degradation of TG molecules and concomitant resynthesis. **a**, Schematic representation of degradation and resynthesis. Blue lines symbolize FAs, and the black horizontal line the glycerol backbone. TG cycling must at least comprise degradation to DG but can include further degradation to MG or free glycerol. All released FAs are supposed to feed a common pool that becomes activated to acyl-CoAs and can be used for re-acylation. Glycerol is not subject to re-acylation in adipocytes and is released into the medium. **b**, Upon cycling, TGs with more than one labelled FA (red lines) will equilibrate their label with the unlabelled (blue) pool, shifting the label from multi- to mono-labelled species. With the dedicated technology developed in the present study, this process can be followed with molecular resolution over time.

Limitations in experimental technology are a major obstacle to a better understanding of TG/FA cycling. When using conventional isotope labelling, the accurate and direct determination of cycling rate and pathways is a challenge for metabolic tracing, because educts and products may become indistinguishable already after one round of cycling (Fig. 1a). Therefore, the existing methods to study TG/FA cycling are rather indirect. Ratiometric determinations of [^3^H]FA and [^14^C]glucose incorporation with parallel determination of glycerol release^10^ or similar strategies with stable isotopes^3^ gave information on the degree of FA esterification and re-esterification from which cycling could be deduced at least in relative terms. Other methods use [^2^H]H_2_O or [^18^O]H_2_O labelling. Enrichment of these isotopes in TG delivered information on total synthesis and turnover of TG^11^. Yet, a direct tracing experiment to show that a defined cellular TG pool 1 would give rise to a new pool 2 by reusing the FAs of pool 1, as illustrated in Fig. 1b, is missing. This would require a labelling experiment with a comprehensive multiparallel tracing of all possible labelled TG molecules, which is an enormous technical challenge. We have recently developed a tracing technology based on alkyne-labelled FAs with click-chemistry reporters and mass spectrometry (MS)^12,13^. It combines all features necessary for the study of TG/FA cycling: high sensitivity, unequivocal specificity and, in particular, easy discrimination of single- and multi-labelled lipid species^12^.

In the following, we will apply this technology to provide direct evidence for TG/FA cycling, give an estimation of the half-life of TG in the cycling process and demonstrate that TG cycling is necessary for homeostasis of the stored FA pool.

## Results

### Multi-labelling strategy

**Fig. 2:**
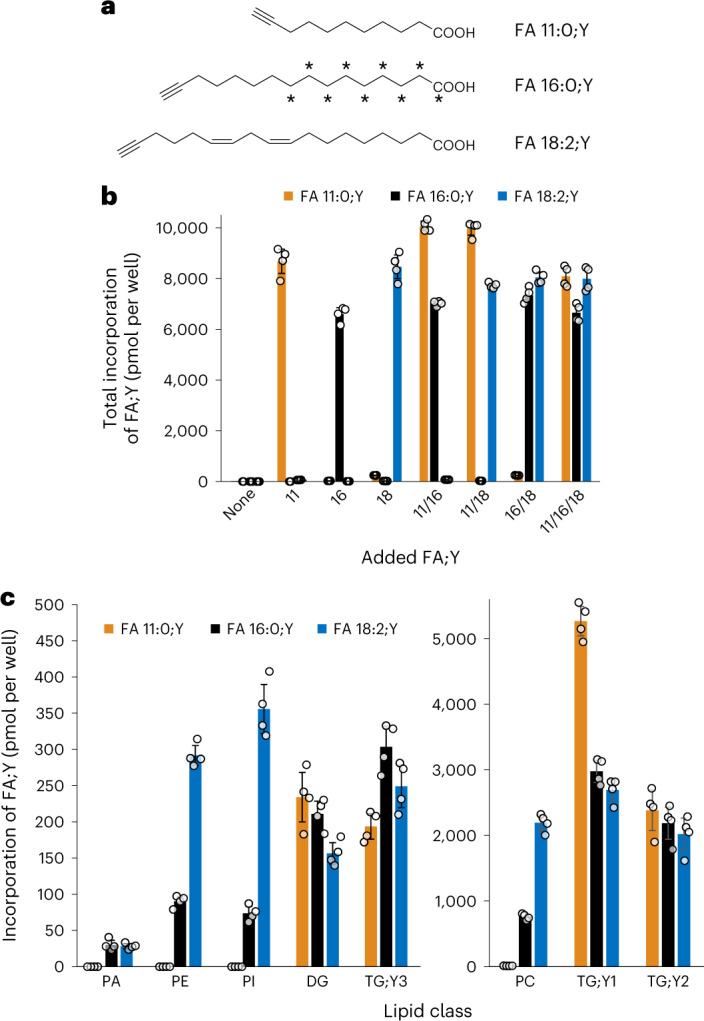
Total incorporation and lipid class preferences of FA;Y in 3T3-L1 adipocytes. **a**, Structures of the FA;Ys used for labelling. **b**, Incorporation of FA;Y per lipid class for three different input FA;Ys. Numbers are pmol per lipid class per well. **c**, Total incorporated FA;Y in major lipid classes upon labelling with the 11/16/18 combination as described in Fig. 1b. Numbers are pmol of respective FA;Y per well. All values are average ± s.d., *n* = 4.

**Extended Data Fig. 1:**
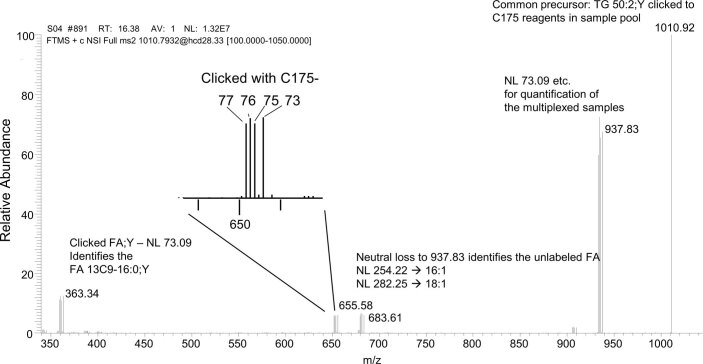
Typical MS2 fragmentation pattern of a single-labeled TG;Y1 and its interpretation. The example is a TG 50:2;Y1 containing FA ^13^C_9_-16:0;Y from a multiplexed sample. After click reaction of individual samples with one of the related C175-7x reagents (Thiele et al., 2019), the pooled samples were analyzed by MS2 of the precursor peak at *m/z* 1010.92. This common precursor undergoes fragmentation by neutral loss of the four isotopic trialkylamines to give the peak series at *m/z* 937.83. Additional neutral loss of fatty acids by elimination gives rise to two peak series at *m/z* 683.61 and 655.58. The difference to the series at 937.83 identifies the two unlabeled FAs in the TG molecule. If the TG molecule under study contains two identical unlabeled FAs, only one peak series is seen in this mass range (see Fig. 5b,c as an example). The inset shows the typical multiplex peak structure produced by the trialkylamine losses of 73, 75, 76 and 77 Da that allows clear assignment to the individual samples in the multiplex pool. The peak series at *m/z* 363.34 is the signal of the click-reacted FA;Y after the neutral loss of 73.09 that was released as a free FA from the TG molecule. This peak series contains information whether the original input FA;Y was used for TG synthesis in unchanged form or was subject to prior modification, for example by elongation, desaturation or partial degradation.

In a conventional tracing experiment, a single-labelled substance is added to a system and its metabolites are followed over time, with a clear logical assignment of labelled metabolites to the original label. However, adipocytes are exposed to the diverse mixture of FAs that originates from the food, with a wide range of length and double bond numbers that form mixed species in the stored TGs. To trace TG synthesis and turnover, an equally diverse set of different FAs should be used as tracers, which would subsequently require unequivocal identification of the label in each possible metabolite. Consequently, the maximum complexity of the labelling mixture is limited by the possible analytical discrimination. To cover a wide range of length and degree of unsaturation with just three compounds, we combine a saturated medium-chain alkyne-FA, a saturated long-chain alkyne-FA and a polyunsaturated long-chain alkyne-FA (alkyne-PUFA). To unequivocally distinguish the three FAs during simultaneous tracing, we use one odd- and one even-numbered alkyne-FA in combination with a third alkyne-FA that additionally carries a stable isotope label. The former two, when metabolically combined with endogenous even-numbered FAs, would lead to distinguishable odd- and even-numbered metabolites, respectively. Metabolites of the third tracer could easily be discriminated by the isotope label. Accordingly, we combined the odd-chain medium FA 11:0;Y (Fig. 2a; for nomenclature of alkyne lipids, see Methods) with the even-chain alkyne-PUFA 18:2;Y and the isotope-labelled saturated long-chain FA ^13^C_9_-16:0;Y. We further combine this threefold multi-labelling with the previously established fourfold sample multiplexing strategy^12^. This saves time and costs while improving the comparability of data, resulting in comprehensive qualitative and quantitative characterization of labelled lipid species (Extended Data Fig. 1).

**Extended Data Fig. 2:**
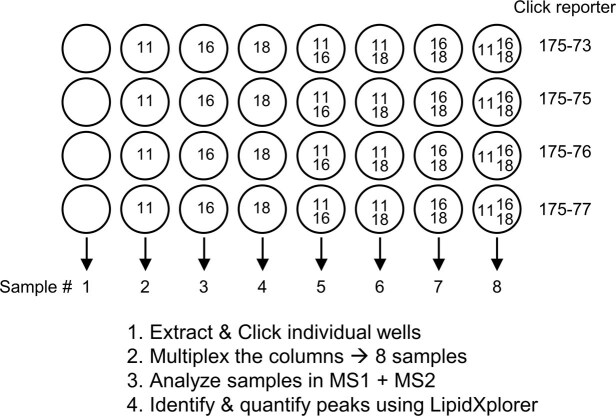
Design and work-flow of the calibration experiment. Circles symbolize the wells of the 24-well plate, containing differentiated 3T3-L1 adipocytes. Numbers 11/16/18 refer to the above input FA;Y used at 50 µM for 2 h. After incubation, the media were removed, cells washed and lipids extracted in the presence of internal standards. Extracted lipids were click-reacted with the C175-7x reporter molecules indicated at the right. After the reaction, samples were pooled such that one pool contained the four multiplexed replicates with identical input but different click reporters, which were then analyzed by MS1 and MS2, followed by data processing using LipidXplorer (Herzog et al., 2011) software.

**Extended Data Fig. 3:**
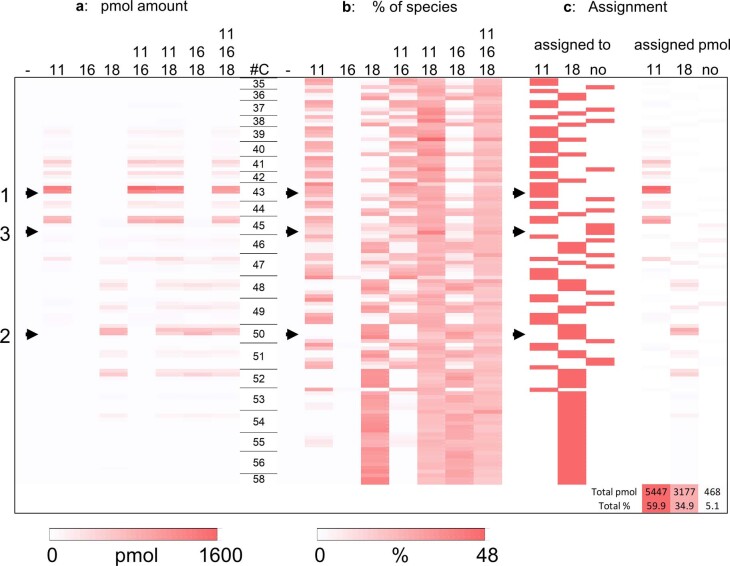
Assignment of single-labeled TG;Y1 species to the input FA 11:0;Y or FA 18:2;Y of the calibration experiment using 3T3-L1 adipocytes. The diagram shows all labeled TG;Y1 species containing FA 11:0;Y or FA 18:2;Y from 35-58 carbons (#C) in the FA chains, with pmol **(a)** or relative **(b)** amounts color-encoded. Each row represents one species. In the first step, each species was analyzed whether it shows the expected typical labeling pattern for FA 11:0;Y (for example **1** →) or FA 18:2;Y (**2** →) or a mixed pattern (**3** →). In the second step, species were assigned to the respective input FA;Y or were not assigned **(c)**. The pmol amount of these assignments taken from the triple labeled samples are summed up at the bottom right, indicating that about 95% of the labeled material was assigned. Note that, against the original expectations, numerous even-numbered species were assigned to FA 11:0;Y and numerous odd-numbered species to FA 18:2;Y. The reason for that is the relatively high abundance of endogenous odd-numbered FAs, mostly 15:0, 15:1, 17:0 and 17:1, in 3T3-L1 adipocytes (Crown et al., 2015). Numbers are encoded by intensity of color (min-max per data block), derived from averages, n = 4.

**Extended Data Fig. 4:**
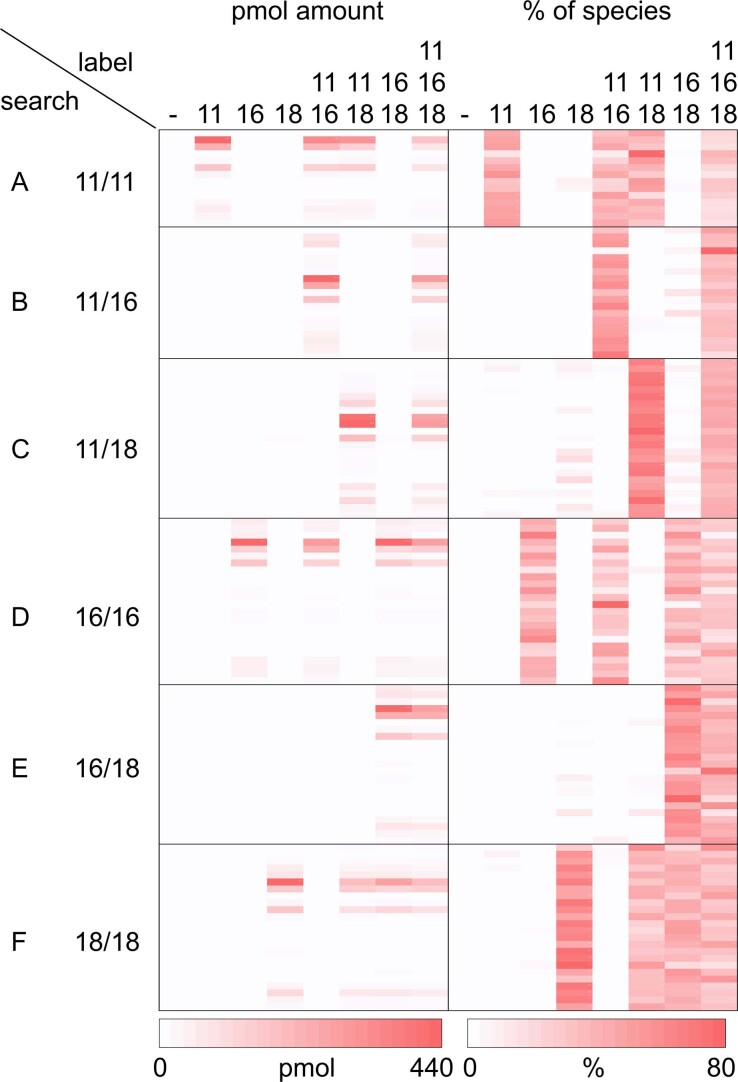
Assignment of double-labeled TG;Y2 species to the input FA;Y in the calibration experiment using 3T3-L1 adipocytes. Picomole (left) and relative (right) amounts of 100 double-labeled TG;Y2 are shown with each block A-F representing one combination of FA;Y in the species search algorithms and each column representing one combination of input FA;Y as indicated at the top. Each row stands for one defined TG;Y2 species. Numbers are encoded by intensity of color (min-max per data block), derived from averages, n = 4.

To establish the separation of MS signals originating from the different FA;Ys and to study the mutual influence of the three FA;Ys, we labelled differentiated 3T3-L1 adipocytes with the three FA;Ys individually and in all possible combinations (Extended Data Fig. 2), as in the following, called the ‘calibration experiment’. Next, we analysed the distribution pattern of each single identified species over the eight labelling combinations and assigned the species to the respective alkyne-FA. The procedure is illustrated and explained in more detail in Extended Data Fig. 3. The assignments from the calibration experiment were translated into LipidXplorer molecular fragment query language (mfql) search files which were optimized to reach a maximum coverage of assignments with parallel exclusion of the ambiguous species. This strategy was expanded to analyse and assign other lipid classes, including multi-labelled species that contain more than one labelled FA. Importantly, also for double-labelled TG;Y2, a clean separation of signals was achieved (Extended Data Fig. 4). The final full analysis covered eight lipid classes, representing the quantitatively relevant products of FA assimilation, that is, sterol ester (CE), ceramide (Cer), diglyceride (DG), phosphatidic acid (PA), phosphatidyl choline (PC), phosphatidyl ethanolamine (PE), phosphatidyl inositol (PI) and TG. This analysis included both single- and multi-labelled species. The latter was particularly important for the labelled TG;Yn. A mixture of 16 labelled internal standards enabled quantification of pmol amounts. After optimization of the required 131 mfql search files, the run time of a multi-labelling analysis was only 5–10 min, depending on the number of samples and the hardware performance. For the calibration experiment, the analysis delivered 406 labelled lipid species. The labelled TGs were dominant, with 127 single-labelled, 128 double-labelled and 10 triple-labelled species, followed by labelled PCs (46 species) and DGs (40 species). All three alkyne-FA labels were well represented, with 121 species for FA 11:0;Y, 181 for FA 16:0;Y and 179 for FA 18:2;Y. Since each sample was fourfold multiplexed, a single sample analysed on the MS instrument delivered up to 1,600 labelled species, each unequivocally identified and quantified.

All three alkyne FAs (Fig. 2a) were incorporated in similar amounts in the cells (Fig. 2b), independent of whether they were applied individually or in combinations. This indicated that the FA uptake and esterification machinery was not saturated in our experiment. The clean separation of the products of the three FA;Ys, as evidenced by the quantitative pattern of identifications in Fig. 2b, is a prerequisite of the subsequent analysis of label distribution in the triple-labelled cells (Fig. 2c). The medium-chain FA 11:0;Y had a clear preference for TG and DG, while the two long-chain FA;Ys were found both in phospholipids and in neutral lipids. Within the phospholipids, the polyunsaturated FA 18:2;Y was more abundant than the saturated FA 16:0;Y. Within the TGs, FA 11:0;Y preferred the single-labelled TGs, while the other FA;Ys were equally found in single- and double-labelled TG species.

### Time-resolved analysis

**Fig. 3:**
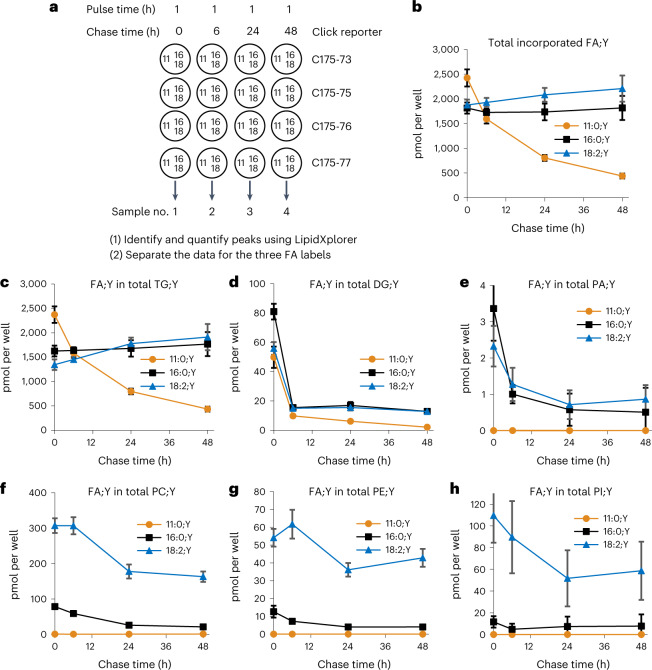
Distribution over time of the three FA;Ys in the major lipid classes of 3T3-L1 adipocytes in a pulse-chase experiment. **a**, Basic design of a pulse-chase experiment. Circles symbolize the wells of the 24-well plate, containing differentiated 3T3-L1 adipocytes. Numbers 11/16/18 refer to the C-count of the input FA;Y (Fig. 2) used at 50 µM for 1 h. After incubation, the media were removed and replaced with chase media for the times indicated. Thereafter, cells were washed and lipids extracted in the presence of internal standards. Extracted lipids were click-reacted with the C175-7x reporter molecules indicated at the right, and samples pooled and subsequently analysed by multiplexed MS1/MS2. Identified labelled species were quantified, summed up and plotted over chase time for the total (**b**) and separately for TG (**c**), DG (**d**), PA (**e**), PC (**f**), PE (**g**) and PI (**h**). In panels **b**–**e**, the sums of single- and multi-labelled species are shown. For PE (**g**), no double-labelled species were detected; for PI (**h**), there were very weak signals of double-labelled species that could not be quantified due to lack of a PI;Y2 internal standard. All values are average ± s.d., *n* = 11–12. Error bars smaller than symbol size are omitted for clarity.

With the new method, we performed a time-resolved analysis of lipid turnover in 3T3-L1 adipocytes. Cells were labelled for 1 h with all three input FA;Ys, each at 50 µM, followed by chase times of 0, 6, 24 and 48 h. After the chase, the cellular lipids were extracted and analysed for labelled species. The complete quantified set of identified lipids together with some annotations can be found in Supplementary Data 1 in Excel format. The total amounts of label in the major lipid classes were determined (Fig. 3). Total incorporated FA;Y was about constant over time for FA 16:0;Y, showed a moderate increase for FA 18:2;Y, but showed a strong decrease for the medium-chain FA 11:0;Y (Fig. 3b).

Note that these and the following numbers comprise the original FA;Y as well as possible metabolites of elongation, desaturation or partial degradation reactions. As expected for an adipocyte model, most of the labelled FAs were incorporated into TG (Fig. 3c). Consistent with its loss in the total, FA 11:0;Y in TG;Y also showed a strong decrease, while the amount of long-chain FA;Y in TG;Y stayed constant (FA 16:0;Y) or increased (FA 18:2;Y) during the chase. About one-half of this increase could be explained by the increase of the total (Fig. 3b), and most of the rest by a flux of FA;Y from labelled DG (Fig. 3d) and PC (Fig. 3f) to labelled TG.

### TG cycling

**Fig. 4:**
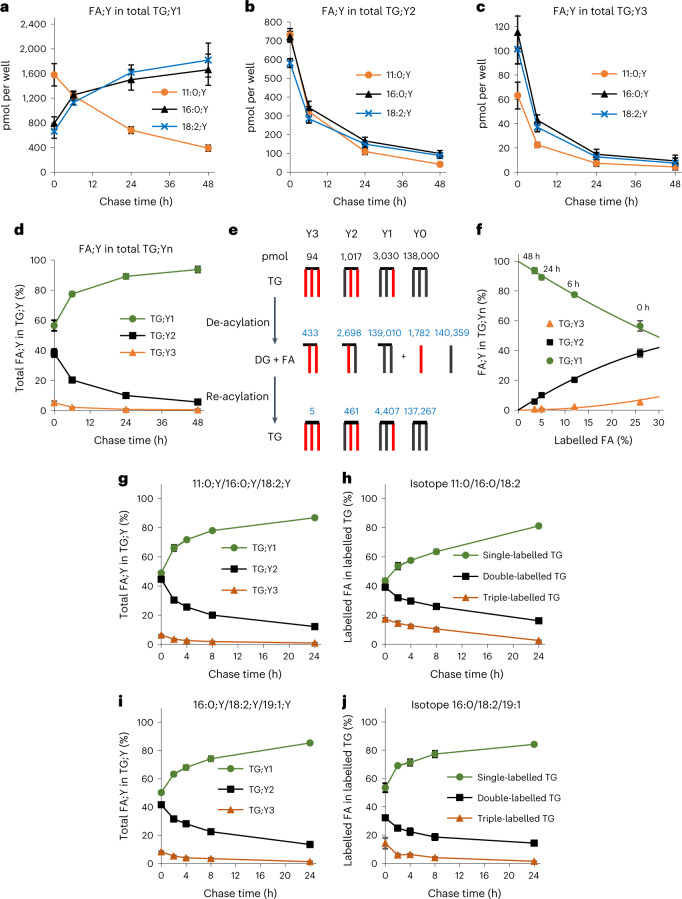
Kinetics of TG turnover and TG cycling in 3T3-L1 adipocytes. **a**–**c**, Time course of the amounts of the three input FA;Ys in single- (**a**), double- (**b**) and triple-labelled (**c**) TG;Yn in the pulse-chase experiment. **d**, Relative percentage distribution of the total FA;Y over the labelled classes of TG;Yn. **e**, Illustration of TG cycling and its effect on the distribution of label between single- and multi-labelled pools. The black numbers are pmol of the respective molecular forms at timepoint 0 derived from the data in panels **a**–**d** and represent the sum of all three FA;Ys. The amount of unlabelled TG;Y0 was also measured in the same samples. The blue numbers are predicted values for one cycle of TG de-acylation followed by stochastic re-acylation. Note that this calculation assumes discrete steps during the cycle, which in reality is not the case since degradation and resynthesis are simultaneous. **f**, The curves show the theoretical calculated binominal label distribution within the pools of TG;Y1, TG;Y2 and TG;Y3 as a function of the fraction of FA;Y of all FAs (unlabelled and labelled; three per TG molecule) in the total pool of TG;Yn. The symbols are the measured values derived from the experiment. The position of the symbols and the corresponding numbers 3.5%, 5%, 12% and 26% indicate the point of best fit of the measured data to the calculated curves. All measured data values were normalized to total FA;Y in TG;Y to compensate for its variations. **g**–**k**, Using the data representation as in panel **d**, TG cycling in separate pulse-chase experiments is compared. All experiments were performed with shorter maximal chase time of 24 h and shorter minimal chase times of 2 h and 4 h. FA label combinations are FA 11:0/16:0/18:2 with alkyne (**g**) or isotope labeling (**h**) and 16:0/18:2/19:1 with alkyne (**i**) or isotope labeling (**j**), as indicated in the panels. All data are average ± s.d., *n* = 11–12 (**g**–**j**, *n* = 4). Error bars smaller than symbol size are omitted for clarity.

**Extended Data Fig. 5:**
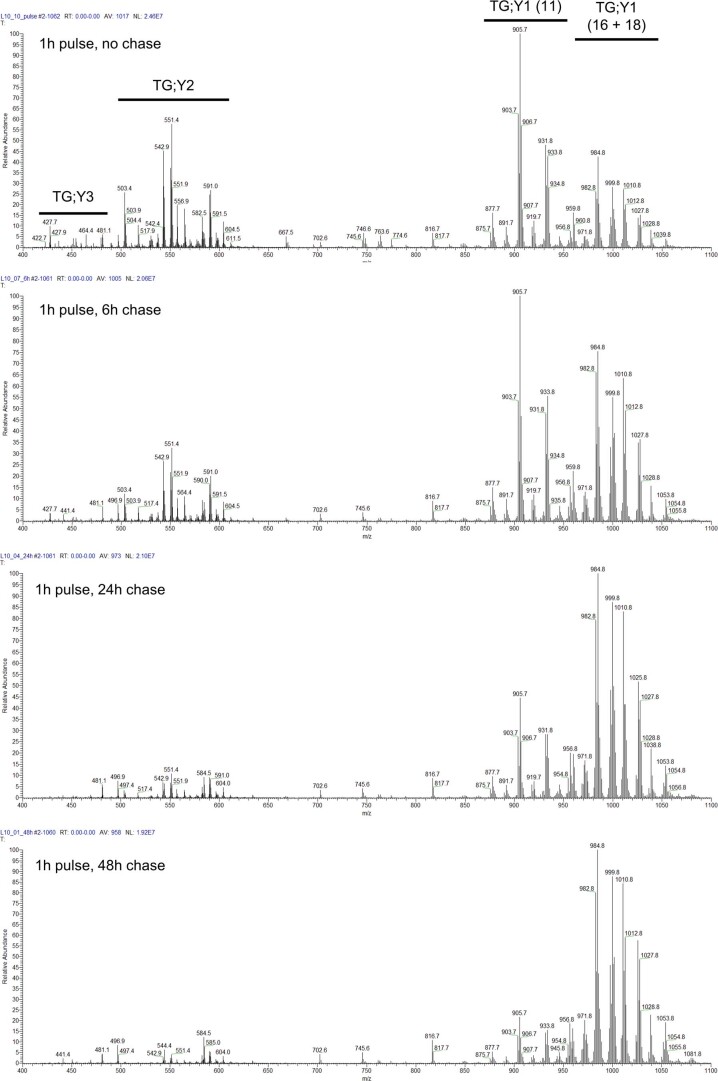
Triglyceride cycling visualized by original neutral loss mass spectra. The four spectra are neutral loss *m/z* 73.1 spectra that show the neutral lipids labeled with C175-73 for pulse and chase times as indicated in the panels. In the top panel, the spectra regions that contain peaks for TG;Y1, TG;Y2 and TG;Y3 are indicated with black bars. Please note the gradual signal decrease of TG;Y3 and TG;Y2 and the parallel increase of TG;Y1. Within the TG;Y1, the selective loss of species containing FA 11:0;Y can clearly be seen.

**Extended Data Fig. 6:**
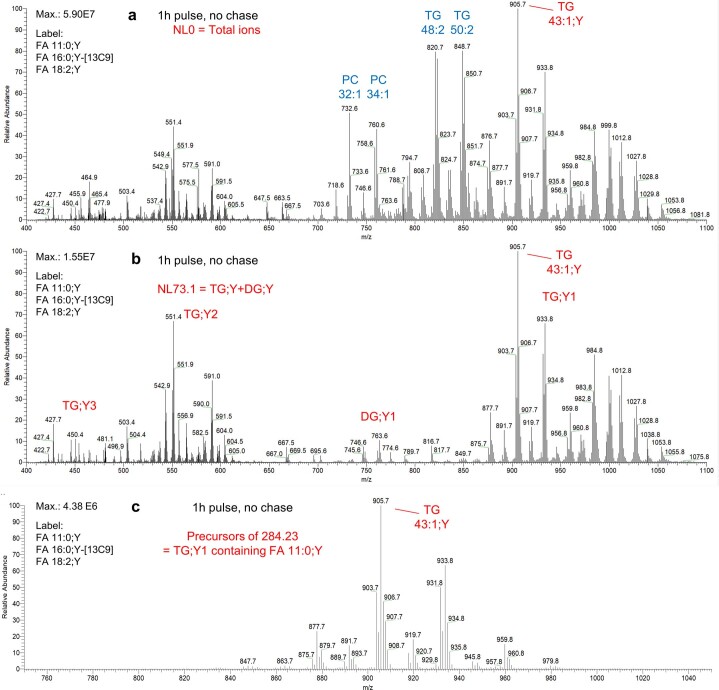
Identification of labeled TG;Y in alkyne labeling experiments. 3T3-L1 cells were labeled with alkyne FA (11:0;Y, 16:0;Y, 18:2;Y, each at 50 µM) for 1 h, and lipid extracts were clicked with C175-7x reagents. MS2 spectra were recorded and, using the ion map function of the Themo Xcalibur software, fragmentation spectra were reconstructed. **Panel a** is a MS2 spectrum of neutral loss of 0 Da. In these experiments, fragmentation is at three different energies, the lowest of which does not lead to fragmentation. Therefore, all MS2 spectra contain a strong peak of the unfragmented precursor ion, which is the NL 0 peak. Major endogenous unlabeled species are labeled in blue, alkyne-labeled species in red. Effectively, **panel a** is a total MS1 spectrum. **Panel b** is the neutral loss *m/z* 73.1 spectrum that shows the alkyne-labeled neutral lipids clicked to C175-73. **Panel c** shows the precursor ion spectrum for the fragment *m/z* 284.23, which is [FA 11:0;Y + (C175-73) – NL 73.1], specific for TG;Y1 containing FA 11:0;Y.

**Extended Data Fig. 7:**
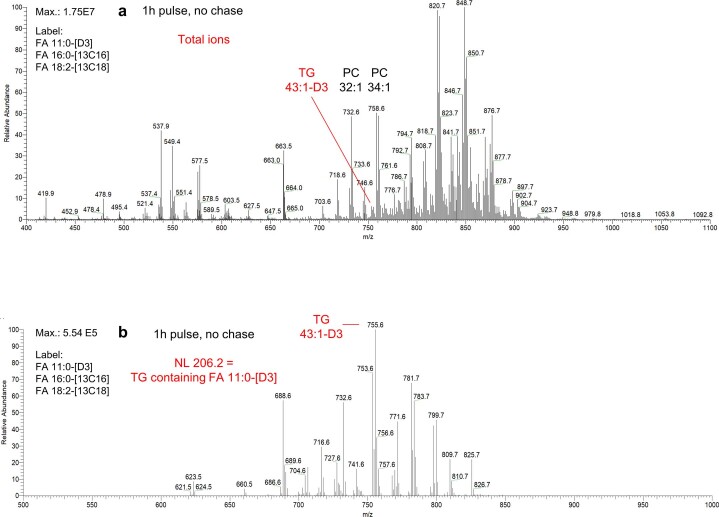
Identification of labeled TG;Y in heavy isotope-FA labeling experiments. 3T3-L1 cells were labeled with heavy-isotope labeled FA (11:0, 16:0, 18:2, each at 50 µM) for 1 h, and lipids extracted. Spectra were recorded and, using the ion map function of the Themo Xcalibur software, fragmentation spectra were reconstructed. **Panel a** is the MS2 spectrum of neutral loss of 0 Da, equivalent to a total MS1 spectrum. **Panel b** shows the neutral loss spectrum for the fragment *m/z* 206.2, which is FA 11:0[D3] + NH_3_, specific for TG containing FA 11:0[D3]. Comment: It is obvious that the isotope labeling data in Extended Data Fig. 7 do not reach the quality of the click-labeling in the corresponding Extended Data Fig. 6. The labeled peaks in the isotope labeling tend to disappear in the background, the intensity of the NL spectrum in **panel b** is only 12 % of that in the corresponding Extended Data Fig. 6c, even though this is actually a multiplex sample that contains the same information four times (additional signals at *m/z* NL 75.1, 76.1 and 77.1). Further, the data in Extended Data Fig. 7b are more complex than those in Extended Data Fig. 6c, because they contain not only the single-labeled species but also all the combinations of FA 11:0[D3] with itself and the other labeled fatty acids. In contrast, Extended Data Fig. 6c is a clean spectrum of TG;Y1 with FA 11:0;Y, because double- and triple-labeled species are at different ranges of the spectrum and show different fragmentation preferences.

Next, we analysed the distribution of FA;Y within the three TG;Yn pools by parallel tracing of >200 species. Over the chase period, we found a massive increase in single-labelled TG;Y1 species, in particular those containing long-chain FA;Ys (Fig. 4a), with a concomitant strong decrease of all three FA;Ys in double- and triple-labelled TG;Yn species (Fig. 4b,c). This behaviour can also directly be seen in the original spectra that visualize the alkyne-labelled neutral lipids by their neutral loss (NL) of 73.1 m/z (Extended Data Fig. 5). The kinetics of these decreases were different for double- and triple-labelled TG;Yn species. After 6 h, 46.7 ± 4.1% of the initial TG;Y2 was still present (Fig. 4b), in contrast to 36.5 ± 3.5% of the initial TG;Y3 (Fig. 4c). Regarding the decrease in TG;Y3, there was very little relative difference between the three input FA;Ys; regarding TG;Y2, the medium-chain FA;Y decreased slightly faster (residual amount after 6 h: FA 11:0;Y, 44.0%; FA 16:0;Y, 47.6%; FA 18:2;Y, 49.0%) than the two long-chain tracers. For all three FA;Ys together, the data were plotted as the fraction of total FA;Y that is found in TG;Y1, TG;Y2 and TG;Y3 (Fig. 4d). They demonstrate the re-distribution of FA;Y from triple- and double-labelled species towards single-labelled TG;Y1. TG cycling is the only consistent explanation for this observation. The most simple cycle would consist of TG hydrolysis yielding DG + FA and subsequent re-acylation to TG (Fig. 4e). The top row shows the situation after the 1-h labelling period. During the pulse labelling, the input FA;Ys (red) are a major fraction of the total FA that is used for TG synthesis, resulting in a large fraction of multi-labelled TG species. In numbers, the labelled TG;Yn pool contained 94 pmol of TG;Y3, 1,017 pmol of TG;Y2 and 3,030 pmol of TG;Y1 at the end of the pulse, provided as black numbers above the top symbols (Fig. 4e). Further, there is the unlabelled pool of endogenous TG of 138,000 ± 7,000 pmol, as obtained from lipidomics of unlabelled TG in the same samples. Following a theoretical binominal equilibration, the dilution of label can be calculated (blue numbers) for one complete TG → DG → TG cycle, predicting a 55% decrease of TG;Y2 and a 45% increase in TG;Y1, which is close to the situation at the 6-h chase time. Figure 4f shows an alternative way of representing the data. The lines represent the theoretical binominal distribution of total FA;Y over the three classes of TG;Yn, as a function of the fraction of FA;Y in the total FA of the TG pool. For each of the four experimental time points, we now determined the position of the data along the *x* axis that would best fit a binominal distribution. For the pulse alone, this results in a total fraction of FA;Y in total synthesized TG;Yn of 26%. This number indicates that during the labelling period, the labelled FA;Y contributed 26% of all FAs that were acylated to TG. In the absence of TG cycling, the relative amounts of the three labelled TG;Yn species would stay constant over time. However, the experimental data indicate that, with increasing chase time, the labelled FA;Y spreads from the small original pool to increasing pool sizes corresponding to 12%, 5% and 3.5% label content. This cycling-dilution process also explains why TG;Y3 disappeared faster than TG;Y2. Unless TG-degrading enzymes could sense the number of triple bonds in a TG species, for which there is no indication so far, both TG;Y2 and TG;Y3 would be degraded at the same rate. The apparent differences in the degradation kinetics are due to the different statistics of the re-acylation of DG;Y1 and DG;Y2 to TG;Y2 and TG;Y3, respectively. Quantitatively, these data indicated that the 6-h chase time corresponded to about 1.5 *t*_1/2_, suggesting a *t*_1/2_ of about 4 h. We next asked whether the observed cycling depends on a specific FA label combination and whether the alkyne label as such might be the cause of the cycling phenomenon (Fig. 4g–j). Therefore, we performed a pulse-chase experiment as described above, but with different FA combinations, that is, FA 11:0;Y/16:0;Y/18:2;Y (Fig. 4g) or FA 16:0;Y/18:2;Y/19:1;Y (Fig. 4i). The FA 19:1;Y has previously been used in hepatocytes^12,13^ and is a good analogue of oleic acid. The latter FA combination is a close mimic of the natural FA composition of the cells under study regarding chain length and double bond count (see Supplementary Table 1 for a detailed analysis of average C-atom and double bond numbers for the labelling experiments shown in Fig. 4g–j). Comparison of data from both FA combinations (Fig. 4g,i) shows that the cycling is independent of the presence of a medium-chain FA and takes place with very similar kinetics. Please note that these experiments have a higher kinetic time resolution and confirm the kinetics of the previous experiment. We also performed analogous pulse-chase experiments using isotope-labelled FA rather than alkyne-labelled FA. Combinations were FA 11:0[D3]/16:0[13C16]/18:2[13C18] (Fig. 4h) and FA 16:0[13C16]/18:2[13C18]/19:1[D8] (Fig. 4j). The analysis of isotope-labelled samples is demanding because isotope labelling lacks the superior analytical sensitivity and specificity hallmarking the alkyne tracers. As an illustration, Extended Data Figs. 6 and 7 show a comparison of primary spectra from the experiments in Fig. 4g,h. Alkyne-labelled TG species separate well from the bulk of unlabelled material (Extended Data Fig. 6a,b), and closer inspection indicates that the major labelled species are the same as the dominant endogenous species (see annotated spectrum in Supplementary Spectrum 1). In contrast, it is unavoidable that the isotope-labelled TGs become part of the crowded region of *m*/*z* 700–900 (Extended Data Fig. 7a). At least the peak of the dominant labelled TG 43:1 could be directly identified in the mixture, but the other species were not visually identifiable. An equivalent of the NL73 search for alkyne-labelled species does not exist for the isotope-labelled species, but the TGs that contained the labelled FA 11:0[D3] could be identified using an NL search for the loss of that FA together with ammonia, that is, at NL *m*/*z* 206.2. Such analysis was performed (Extended Data Fig. 7b) and yielded a reasonable spectrum. An equivalent LipidXplorer search algorithm was then used to identify and quantify the labelled TGs. Corresponding searches were also performed for the other isotope-labelled FAs. Although this is necessary to achieve the required specificity, it means that each group of isotope-labelled TGs is quantified by a different neutral loss, in contrast to the uniform quantification of the alkyne-labelled species, introducing a possible quantitative bias in the data. Finally, we could identify 105 isotope-labelled TG species (versus 270 alkyne-labelled TG species), just enough for calculations for single-, double- and triple-labelled TGs (Fig. 4h,j). These data showed that TG cycling takes place in the same way and with comparable kinetics for isotope- and alkyne-labelled species.

### Metabolism of the FAs

**Fig. 5:**
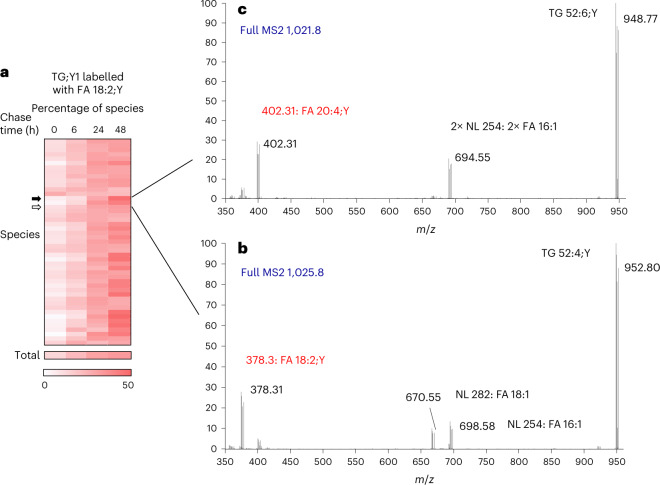
Detection of modified FA;Y assimilated in labelled lipids of 3T3-L1 adipocytes. **a**, Time course of relative abundance of TG;Y1 species upon labelling with FA 18:2;Y. Relative amounts of all TG;Y1 species of the pulse-chase experiment are shown colour-encoded. Each row represents one species. **b**, MS2 analysis of one species (open arrow) at chase time 48 h. The precursor peak at *m*/*z* 1,025.8 is omitted. The four series of fragment peaks indicate the FA composition 16:1_18:1_18:2;Y. **c**, MS2 analysis of one species (closed arrow) at chase time 48 h. The precursor peak at *m*/*z* 1,021.8 is omitted. The three series of fragment peaks indicate the FA composition 16:1_16:1_20:4;Y. Note that in **b** and **c** the actual incorporated FA;Ys are directly identified from the peak masses and printed in red. In **a**, numbers are encoded by intensity of colour (min–max of the entire data block), derived from averages, *n* = 12. Detailed statistical data of this experiment are shown in the Supplementary Table 2.

We next deepened the analysis by advancing to the lipid species level. Analysis of TG;Y1 species after labelling with FA 18:2;Y (Fig. 5a) showed that, within this lipid class, some species were relatively constant (Fig. 5a, open arrow) and others (Fig. 5a, closed arrow) increased more strongly than the general trend (Fig. 5a, total).

**Fig. 6:**
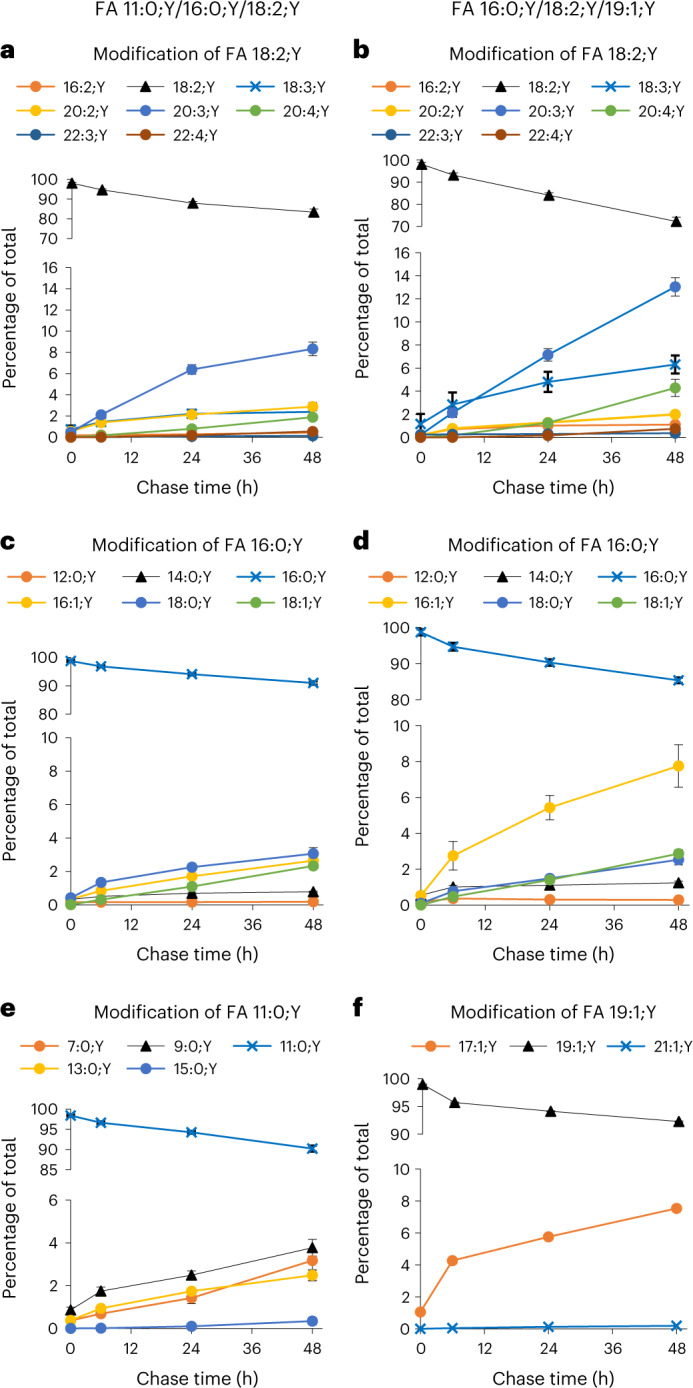
Time course of FA modification of added FA;Y in 3T3-L1 adipocytes. 3T3-L1 cells were labelled with the FA combination 11:0;Y/16:0;Y/18:2;Y (**a**,**c**,**e**) or 16:0;Y/18:2;Y/19:1;Y (**b**,**d**,**f**) for 1 h, followed by chase periods between 6 h and 48 h, as indicated. FA fragment peaks as illustrated in Fig. 5b,c were detected, identified and quantified in all single-labelled TG;Y1 species. Diagrams show the fraction of each metabolite at the four time points. Metabolites of FA 18:2;Y are shown in panels **a** and **b**, of FA 16:0;Y in panels **c** and **d**, of FA 11:0;Y in panel **e** and of 19:1;Y in panel **f**. All numbers are normalized to the total of the respective FA;Y in TG;Y1 at the respective chase time to compensate for changes in total TG;Y1. Ordinates are split to show the decrease of the input FA;Ys and the increase of their metabolites in one panel. All numbers are average ± s.d., *n* = 11–12. Error bars smaller than symbol size are omitted for clarity.

**Extended Data Fig. 8:**
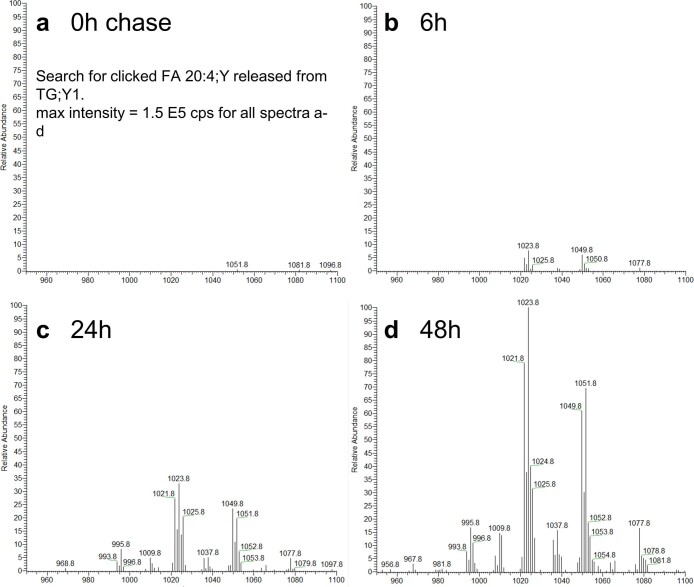
Time course of appearance of TG;Y1 containing FA 20:4;Y. Raw MS2 spectra from samples of the pulse-chase experiment were searched for the fragment *m/z* 398.28, tolerance 0.01 Da, that is characteristic for the presence of FA 20:4;Y in TG;Y1. The panels show the resulting precursor ion spectra; all are plotted at the same scale for maximum intensity. Major species are: TG 52:6;Y at *m/z* 1021.8, TG 52:5;Y at *m/z* 1023.8, TG 54:6;Y at *m/z* 1049.8, TG 54:5;Y *m/z* 1051.8.

**Extended Data Fig. 9:**
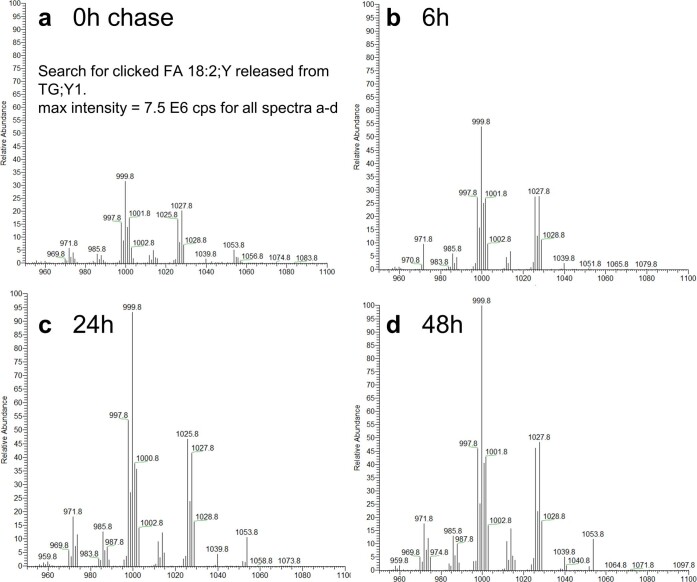
Time course of appearance of TG;Y1 containing FA 18:2;Y. Raw MS2 spectra from samples of the pulse-chase experiment were searched for the fragment *m/z* 374.27, tolerance 0.01 Da, that is characteristic for the presence of FA 18:2;Y in TG;Y1. The panels show the resulting precursor ion spectra; all are plotted at the same scale for maximum intensity. Major species are: TG 50:4;Y at *m/z* 997.8, TG 50:3;Y at *m/z* 999.8, TG 50:2;Y at *m/z* 1001.8, TG 52:4;Y at *m/z* 1025.8, TG 52:3;Y at *m/z* 1027.8.

**Extended Data Fig. 10:**
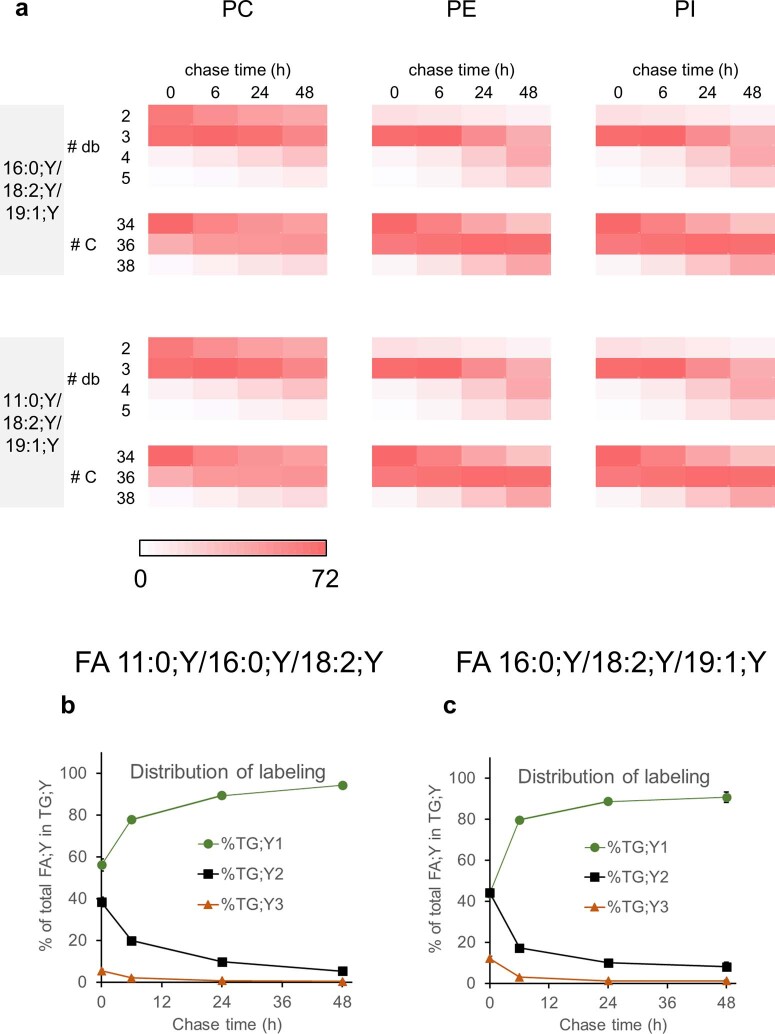
Rate of overall TG cycling and desaturation and chain length of phospholipids labeled with FA 18:2;Y. Differentiated 3T3-L1 adipocytes were labeled with FA combinations as indicated for 1 h followed by chase periods as indicated. Cells were washed and lipids extracted, click-reacted with the C175-7x reporters and analyzed by multiplexed MS1/MS2. **a**, Abundance of labeled species containing 2-5 double bonds (# db) and 34-38 C atoms (# C) in the FA chains were calculated and are expressed as percent of the total of the 18:2;Y-labeled lipids of the respective phospholipid class. Numbers are encoded by intensity of color, derived from averages, n = 12. **b,c**, Relative percent distribution of the total FA;Y over the labeled classes of TG;Yn. Data are avg. +/- StdDev, n = 11-12. Note that data in **panel b** are identical to those in Fig. 4d.

By MS2 analysis, the constant species (Fig. 5b) was identified as TG 52:4;Y, containing unlabelled FAs 16:1 and 18:1 together with FA 18:2;Y. In contrast, the increasing species (Fig. 5c) was identified as TG 52:6;Y, containing two unlabelled FAs 16:1 and a labelled FA 20:4;Y, indicating metabolism of the labelling FA;Y itself by elongation and desaturation. This was confirmed by direct searches in the original spectral data (Extended Data Fig. 8 for FA 20:4;Y and Extended Data Fig. 9 for FA 18:2;Y) and a detailed statistical analysis (Supplementary Table 2). Therefore, using dedicated LipidXplorer mfql files, we systematically searched the MS2 spectra of all single-labelled TG;Y1 species for metabolically processed FA;Ys, all of which were clearly identified and quantified. TG;Y1 was chosen because (1) its spectra contained the clearest information about FA;Y1 identity and (2) it contained the majority of labelled material, particularly at later chase times. Data from both FA combinations, 11:0;Y/16:0;Y/18:2;Y and 16:0;Y/18:2;Y/19:1;Y, were analysed. The results, normalized to percentage of the total in TG;Y1, showed that in both FA combinations the strongest modification occurred in the polyunsaturated FA 18:2;Y (Fig. 6a,b), which was elongated and desaturated to give FA 18:3;Y, FA 20:3;Y and FA 20:4;Y as the most prominent products. The saturated FA 16:0;Y was modified slightly less (Fig. 6c,d), mostly by desaturation and elongation, giving rise to FA 16:1;Y and FA 18:1;Y. FA 11:0;Y was partially degraded, yielding FA 7:0;Y and 9:0;Y, and partially elongated to FA 13:0;Y and 15:0;Y (Fig. 6e). FA 19:1;Y was partially degraded to FA 17:1;Y and to a very small extent also elongated to FA 21:1;Y. In general, modification was stronger in the FA combination 16:0;Y/18:2;Y/19:1;Y, which correlated with a faster overall TG cycling in this experiment (Extended Data Fig. 10b,c).

The observed increase in double bonds and chain length was not confined to the TG;Y1. In the phospholipids, the conversion of FA 18:2;Y to FA 20:4;Y cannot be traced as directly as in the TGs because the phospholipids do not release a labelled FA;Y fragment in MS2. Yet, the numbers of side chain C-atoms and of double bonds steadily increased for the three major phospholipid classes, with increasing chase times in both FA combinations (Extended Data Fig. 10a). This finding rules out that the increase of FA 20:4;Y in the TG;Y1 would be fuelled by FA 20:4;Y liberated from the PC;Y1 pool.

**Fig. 7:**
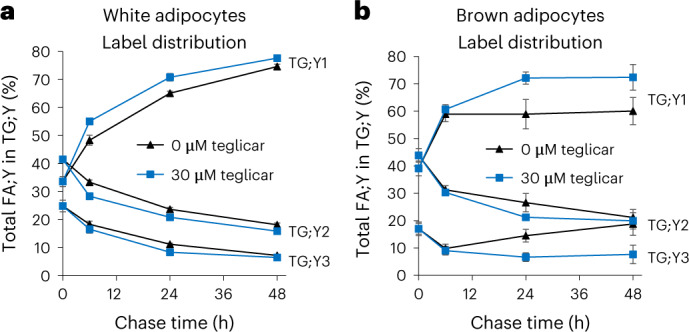
TG cycling in white and brown adipocytes. **a**,**b**, White (**a**) and brown (**b**) adipocytes were labelled with the FA combination 16:0;Y/18:2;Y/19:1;Y for 1 h, followed by chase periods between 6 h and 48 h, as indicated. The diagrams show the distribution of the sum of the three FA;Ys over the TG;Y1, TG;Y2 and TG;Y3 pools, normalized to the total amount of FA;Y in the entire TG;Yn pool. All values are average ± s.d., *n* = 4 (*n* = 12 for chase time 0 h). Blue symbols indicate the presence of the CPT1 inhibitor, teglicar, during the chase periods, and black symbols its absence.

Finally, we performed pulse-chase experiments in murine primary white and brown adipocytes. In both systems, there clearly was TG cycling with rates that were comparable to those found in 3T3-L1 adipocytes (Fig. 7, black triangles). Partial inhibition of beta-oxidation using 30 µM of the CPT1 inhibitor teglicar^14^, whose half-maximum inhibitory concentration (IC_50_) value was determined as 40 µM (ref. ^15^), resulted in an apparent increase of cycling rates (Fig. 7, blue squares) in both adipocyte types. This observation is consistent with the idea that cycling requires both the initial cleavage of TG and the re-synthesis of a TG molecule. If the initially released FA is, however, degraded by beta-oxidation, less labelled TG;Y1 will be produced from unlabelled DG, and consequently the apparent efficiency and rate of cycling will be decreased.

## Discussion

A detailed and quantitative analysis of TG cycling was impossible thus far because of the lack of a suitable method. The multilabel multiplex tracing method that was developed for this project enabled such study, as presented here. The employed strategy for label discrimination using three alkyne-FA tracers (odd/even FA;Y plus one isotope-labelled FA;Y) worked well, even in the challenging system of adipocytes with a considerable background of endogenous odd FAs^16^. The considerable length difference of seven carbons between the odd and even FA;Ys proved helpful for discrimination of labelled species. The ^13^C_9_-labelling of the third FA;Y excluded any relevant interference with the two other FA;Ys. For even more difficult biological systems or for future addition of a fourth tracer, the preparation of other isotope-labelled FA;Ys, for example, by incorporation of some D atoms into a monounsaturated FA;Y, would be an interesting option. Importantly, our methodology is not limited to particular FA combinations or cell types. Apart from the 11:0/16:0/18:2 and 16:0/18:2/19:1 tracers of this study, we have also successfully tested the combination 12:0/16:0/19:1 in adipocytes as well as all these combinations in freshly isolated hepatocytes.

The direct demonstration of TG cycling in 3T3-L1 adipocytes opens several points for discussion. The first is whether the data would allow other explanations. This can essentially be denied. The crucial argument comes from the observation that within the first 6 h of chase, for both FA 16:0;Y and FA 18:2;Y, a strong increase in single-labelled TG;Y1 is found. The release of label from double- and triple-labelled species is the only source that can explain this increase. Using FA 16:0;Y as an example, there is an increase of label in TG;Y1 of 461 pmol and a matching parallel decrease in TG;Y2 and TG;Y3 by 449 pmol. In contrast, the entire decrease in all other lipid classes sums up to 99 pmol, not sufficient to explain the increase in TG;Y1. The corresponding numbers for 18:2;Y indicate an increase in TG;Y1 by 468 pmol, a combined decrease in TG;Y2 and TG;Y3 by 360 pmol, and in all other lipid classes by 56 pmol. Again, numbers indicate that 76% of the increase in TG;Y1 originates from TG;Y2 and TG;Y3, which is by definition TG cycling.

The second point concerns the pathway of TG cycling. The absolute minimum is a TG/DG cycle as depicted (Figs. 1 and 4e), but there could also be a longer cycle that would involve further degradation to monoacylglycerol (MG) or to free glycerol. In fact, glycerol release by adipose tissue is a classical indicator of TG hydrolysis^17^ and was used to estimate TG cycling^3^. More recent research in both 3T3-L1 cells and primary adipocytes showed that liberated glycerol may also originate from glycolysis^18,19^, questioning the idea that glycerol release is a quantitative indicator of TG hydrolysis. At present, our data cannot differentiate between the possible forms of the TG cycle and also cannot indicate the identity of the enzymatic activities involved. The fact that the data nicely match a binominal label distribution indicates the stochastic nature of the process. For deeper mechanistic information, more data at shorter chase periods together with inhibitor and gene ablation studies directed against candidate enzymes are needed. Such data should be fed into mathematical modelling which must include not only the TG;Yn, but also labelled DG;Yn, MG;Y, PA;Y and PC;Y.

A third point worth discussing is the speed and the energetic costs of the cycle. From the degradation of TG;Y3, we can estimate a half-life of 4 h for TG in a TG/DG cycle. With that rate, and ignoring the issue of positional specificity of cycle reactions, each FA in TG would be turned over once a day, with energetic costs of 1 ATP per day. With a net energy content of 106 ATP per molecule of palmitate, the storage of TG would cost about 1% of its energy content per day in 3T3-L1 adipocytes. This seems to be a large number when transferred to adipose tissue, because it would mean that a 50-kg fat depot would shrink at a rate of 0.5 kg per day just by TG cycling. It is likely that in white adipose tissue with its larger lipid droplets, TG cycling occurs at lower rates than in 3T3-L1 cells which have droplets of typically 5–10-µm diameter, as some of the relevant metabolic reactions occur at the droplet surface.

Finally, the question of the biological advantages of cycling over a static storage needs to be discussed. The classical textbook argument for substrate cycles is the improved regulatory flexibility by increasing the amplitude of regulation of a pair of counteracting enzymes over a single one acting unidirectionally. This appears to be true also for TG cycling, since a major part of reduced TG consumption after finishing physical activity can be attributed to increased re-esterification instead of reduced ester hydrolysis^3^. A further function may lie in the UCP1-independent thermogenesis in white adipose tissue^20,21^.

Our data point to a third important aspect: the composition of the stored FA pool in the TGs. The data show that each of the three labelling FAs has a specific fate: a majority of 82% of incorporated medium-chain FA 11:0;Y was lost from the cells within 48 h, presumably by preferential oxidation. About 10% of the remainder were modified, mostly by elongation and desaturation, yielding FA 15:1;Y and 17:1;Y. Only 16% of the original FA 11:0;Y was still present after 48 h. After the same time, 100 ± 13% of alkyne palmitic acid 16:0;Y was still present, and 88% thereof remained in its original form. Modifications produced mostly desaturated FA 16:1;Y and 18:1;Y. The PUFA 18:2;Y did not show significant loss of material. About 20–30% of the original FAs were modified, mostly to FA 20:3;Y and 20:4;Y, the equivalent of arachidonic acid and its precursor. In summary, these data show (1) a fast clearance of medium-chain FAs, (2) a slow desaturation of saturated FAs to produce equivalents of palmitoleic and oleic acid, and (3) a metabolism of linoleic acid equivalents towards arachidonic acid. All three processes are potentially beneficial to the cell or organism. Medium-chain FAs are useless or even potentially dangerous as membrane constituents. Their removal eliminates a potential problem and their conversion to long-chain monounsaturated FAs provides useful membrane building blocks. Palmitic acid is an abundant food constituent, but high concentrations of palmitate in lipids correlates with several adverse conditions^22^. Slow desaturation to beneficial monounsaturated FAs would reduce the danger of palmitate accumulation in stored TGs and prevent negative effects of released palmitate upon mobilization of stored fat. Arachidonate is an important FA with unique enzymatics in membrane lipid turnover^23,24^ and serves a key role as a precursor for eicosanoids in inflammatory signalling. Sustained levels of arachidonic acid are critical for mammalian life, as evidenced by many experimental studies in mice and by the absolute requirement of arachidonic acid in infant nutrition^25^. Conversion of the abundant linoleic acid to arachidonic acid in mammals is a complex multistep process active in hepatocytes^26^. Our data suggest that adipose tissue may support other organs in that process.

In the absence of TG cycling, all three FA;Ys would stay entirely unmodified after incorporation into TG, because all the observed FA modifications or degradation pathways need Coenzyme A esters of free FA (FA-CoA) as a substrate. Therefore, ongoing FA modification necessitates the repeated release of FAs from TG in conjunction with their reactivation by acyl-CoA synthetases in the TG cycling pathway. The same logic also applies to the clearance of damaged FAs, for example, by peroxidation, from the TG pool. In a static pool, damaged material would accumulate; TG cycling gives access to damaged FAs for subsequent degradation. In summary, our experiments emphasize the essential function of TG cycling for monitoring and maintaining the composition of the stored FA pool in adipocytes.

## Methods

### Lipid nomenclature

To avoid confusion between normal lipid double bonds and the alkyne label terminal triple bond, we treat the triple bond as a functionalization of the FA and indicate it with a suffix ‘;Y’. This follows the strategy of the LipidMaps nomenclature^27^, which does not yet have a system to indicate triple bonds in the most recent update^28^. The nomenclature used here was discussed and agreed upon with a group of interested scientists who participated in the latest update of the LipidMaps nomenclature and was used already in our recent publications. The Y makes the triple bond visible in the abbreviation and keeps the correct number of double bonds in the abbreviation. This results in FA 18:2;Y for the terminal alkyne-FA with 18 C-atoms and two double bonds, which is used as a linoleic acid equivalent in this study. By that, both the functionalization with a triple bond and the biochemical equivalence to linoleic acid is easily accessible. For lipid classes, we also use the suffix Y, that is, PC;Y and TG;Y indicate phosphatidylcholine and triacylglycerol that contain an alkyne-FA (FA;Y), respectively. The presence of two FA;Ys in one double-labelled TG molecule gives TG;Y2, and that of three FA;Ys in a triple-labelled molecule gives TG;Y3, accordingly. In glycerolipid species names, the ‘Y’ will be placed next to the double bond number, for example, TG 52:3;Y is a very frequent product of labelling with FA 18:2;Y. Accordingly, if the identity of the other FA in the TG;Y is known, the molecule could become TG 18:2;Y_16:0_18:1, and, if positions were known, TG 16:0/18:2;Y/18:1.

### Materials

FA 11:0;Y was obtained from TCI Germany, and FA 18:2;Y was synthesized as described^29^. FA 19:1[D8] was synthesized by Wittig reaction between carboxyoctyl-triphenyphosphonium bromide and decanal[D8]. The latter was prepared from decatetraenal^30^ by deuteration with deuterium gas and Wilkinson’s catalyst in CH_3_OD. ^13^C_9_-FA 16:0;Y was obtained in a multistep chemical synthesis. Briefly, commercial U-^13^C-labelled mixed FA methyl ester was reacted with *cis*-1,4-diacetoxy-2-butene in the presence of nitro-Grela as a metathesis catalyst. From the mixture obtained, ^13^C_9_-11-acetoxyundec-9-enoic acid methyl ester was purified. The double bond was reduced with H_2_/Pd, and the acetylester cleaved with HCl in methanol. The resulting ^13^C_9_ 11-hydroxyundecanoic acid methyl ester was purified and oxidized with pyridinium chlorochromate in dichloromethane to the corresponding aldehyde, 11-oxoundecanoic acid methyl ester. This was coupled with the Wittig reagent obtained from dioxolanylpropyl-triphenylphosphonium bromide. The resulting double bond was reduced with H_2_/Pd. The protecting group on the terminal aldehyde was removed with toluenesulfonic acid in wet acetone to obtain ^13^C_9_-15-oxo-pentadecanoic acid methyl ester. The final terminal triple bond was introduced by reaction with Bestmann–Ohira reagent and the methyl ester cleaved with KOH in tetrahydrofurane/water to obtain the final product at about 100 mg. MS analysis indicated a purity of about 87%. The major impurities are 4–5% each of ^13^C_10_-17:0;Y and ^13^C_12_-19:0;Y. These impurities, likely originating from double bond migrations in the initial metathesis reaction, do not disturb the analysis since MS peaks resulting from them are easily discriminated from the ones that contain ^13^C_9_-16:0;Y.

Internal standards were those used in the previous studies^12,31^ with additional standards to monitor TG;Y3, PA;Y2, PC;Y2 and PE;Y2. To each sample, a mixture containing the internal standards was added (for composition and amounts, see Supplementary Table 3). The internal standards for each lipid class are supplied in different amounts to account for the different abundancies of the respective classes. The aim is to have standard intensities that are in the typical range of sample intensities for the same lipid class.

### Cell culture

3T3-L1 preadipocytes (ATCC CL-173) were cultivated in growth medium (DMEM 4.5 g l^−1^ glucose, 10% FCS plus penicillin/streptomycin), seeded into 48-well plates and induced to differentiate after 24 h by addition of 1 µM rosiglitazone, 10 µg ml^−1^ insulin, 10 µM dexamethasone and 0.5 mM 3-isobutyl-1-methylxanthine for 3 days, followed by culture in growth medium with insulin only for 2–4 days, with media changes every second day. Differentiation was monitored regularly by the appearance of visible lipid droplets in phase-contrast microscopy.

### Isolation and differentiation of primary brown and white adipocytes

Mice were maintained and bred in the Bonn Life and Medical Sciences Institute animal facility. The mice had free access to standard rodent diets and water. Animals were housed in a 12:12 light/dark cycle, at 23 ± 1 °C. All animal studies were performed according to German animal welfare laws. Animal experiments were permitted by the Landesamt für Natur, Umwelt und Verbraucherschutz (LANUV) Nordrhein-Westfalen, Germany, 81-02.04.2021.A166. Primary adipocytes were isolated from 8–12-week-old C57BL/6 mice. Dissected inguinal white adipose tissue and interscapular brown adipose tissue from 2–3 different mice was digested in DMEM (Invitrogen) containing 0.5% BSA and collagenase NB46 at 37 °C, and then centrifuged at 1,000 r.p.m. for 10 min. The resulting pellet was resuspended and filtered using a 100-μm cell strainer. The filtered solution was seeded in a T175 culture flask in DMEM supplemented with 10% FBS, 1% penicillin/streptomycin and 1% amphotericin B (white adipocyte (WA) Growth medium), and kept at 37 °C and 5% CO_2_. At 24 h after seeding, cells were washed with PBS and maintained with WA Growth medium at 37 °C, 5% CO_2_. Medium was changed every other day until cells reached confluency and cells were subsequently cryopreserved.

Cells were plated at a density of 20,000 cells per well in a 48-well plate in growth medium. Cells were grown to confluency in growth medium. Once confluent, preadipocytes were induced during 48 h (day 0 to day +2) by changing the medium to WA Induction medium (DMEM containing 5% FBS, 1% P/S, 1 nM T3, 0.172 µM insulin, 50 mg ml^−1^ l-ascorbate, 1 mM d-biotin, 17 mM pantothenate, 1 µM rosiglitazone, 0.25 µM dexamethasone and 0.5 mM 3-isobutyl-1-methylxantine). From day +2 until day +12, cells were maintained in WA Maintenance medium (DMEM containing 5% FBS, 1% P/S, 1 nM T3, 0.172 µM insulin, 50 mg ml^−1^ l-ascorbate, 1 mM d-biotin, 17 mM pantothenate), refreshing it every other day. Cells were used for experiments on day +12.

### Labelling

For the calibration experiment, culture medium was replaced by growth medium containing FA;Ys as indicated in the figure legends, each at 50 µM concentration, for 1 h. Media were removed and then cells washed and lipids extracted for further analysis. For the pulse-chase experiment, cells were labelled with growth medium containing 50 µM of each of the three FA;Ys or heavy-isotope-labelled FA, both as indicated in the figure legends, for 1 h. Media were removed, cells washed once and then either processed for lipid extraction and analysis (no chase), or fresh growth medium added for the chase times, as indicated. After the chase, media were removed, and cells were washed and processed for extraction and analysis.

### Lipid extraction and click reaction

Cells on multiwell dishes were washed once with warm medium and once with ice-cold PBS and quickly once with 155 mM ammonium acetate, taking care to remove the liquid after the last wash as completely as possible. To each well, 500 µl of extraction mix (490 µl of MeOH/CHCl_3_ 5:1, 10 µl of internal standard mix containing alkyne-labelled standard lipids and a nonalkyne internal standard for TG (TG 50:1[D4])) as indicated above was added and the entire plate was sonicated for 1 min in a bath sonicator. The extracts including the cell remnants were collected into 1.5-ml original Eppendorf tubes and centrifuged at 20,000*g* for 5 min to pellet protein. The supernatants were transferred into fresh tubes. After addition of 400 µl of CHCl_3_ and 600 µl of 1% AcOH in water, samples were shaken for 30 s and centrifuged for 5 min at 20,000*g*. The upper phase was removed, and the lower phase transferred into a fresh tube and dried for 20 min at 45 °C in a speed-vak. CHCl_3_ (8 µl) was added and the tubes briefly vortexed. To each tube, 40 µl of Click mix was added (prepared by mixing 10 µl of 100 mM C175-7x in 50% MeOH (stored as aliquots at −80 °C) with 200 µl of 5 mM Cu(I)AcCN_4_BF_4_ in AcCN and 800 µl of ethanol), followed by sonication for 5 min and incubation at 40 °C for 16 h.

To samples clicked with C175-7x, 100 µl of CHCl_3_ per sample was added and multiplex samples pooled. To the pool, 600 µl of water was added and pools briefly shaken and centrifuged for 2 min at 20,000*g*. The upper phase was removed and the lower phase dried in a speed-vac as above. Spray buffer (2-propanol/methanol/water 8:5:1 + 10 mM ammonium acetate) (200–1,000 µl) was added, the tubes sonicated for 5 min and the dissolved lipids analysed by MS.

### MS

Mass spectra were recorded on a Thermo Q-Exactive Plus spectrometer equipped with a standard heated electrospray ionization (HESI) ion source using direct injection from a Hamilton syringe driven by a syringe pump under the control of the Tune instrument control software. To minimize dead volumes, the syringe port was directly connected to the HESI source without connecting tubings. Samples were sprayed at a flow rate of 10 µl min^−1^ in spray buffer with the following parameters: sheath gas 6, aux gas 2, sweep gas 0, gas heating off, spray voltage positive mode 4.1 kV, ion transfer capillary temperature 280 °C.

MS1 spectra were recorded as segmented scans with windows of *m*/*z* 100 between *m*/*z* 300 and 1,400 for 1.2 min, followed by MS2 spectra by data-independent acquisition for 19 min using inclusion lists from *m*/z 305.373 to 1,400.119 at *m*/*z* 1.0006 intervals, to adapt to the typical mass defects of lipids at the respective masses. Typical scan parameters were, for MS1 scans: automatic gain control target 3 × 10^6^, maximum ion time 800 ms, resolution 280,000, peak mode centroid; for MS2 scans: automatic gain control target 2 × 10^5^, maximum ion time 700 ms, resolution 280,000, no spectral multiplexing, dynamic first mass, isolation window *m*/*z* 0.4, stepped normalized collision energy 10, 35, 40, spectrum data type centroid. In addition, a second scan for double-charged species was performed in the scan range of *m*/*z* 300–700, with MS2 scans from *m*/*z* 300.8052 to 700.0648 at intervals of *m*/*z* 0.5002 and an isolation window of *m*/*z* 0.4. Triple-charged species (TG;Y3 and PC;Y2) were scanned using a targeted inclusion list comprising all theoretical combinations of FA;Y with an isolation window of *m*/*z* 0.3.

### Identification of alkyne-labelled lipids

Raw files were converted to .mzml files using MSConvert and analysed using LipidXplorer^32^. For identification and quantification of labelled lipids, mfql files were written which identify the species by the presence of a peak corresponding to the expected masses of the labelled lipid class combined with the characteristic neutral losses. For details and examples, see the Supplementary information and ref. ^12^. One complete search comprised 44 mfql files for single-charged species, 69 for double-charged and 18 for triple-charged species. For search of FA modification in TG;Y species, another set of 20 mfql files was used. Subsequent data processing was done using standard MS Excel calculation.

### Identification of isotope-labelled TG species

The actual pulse-chase experiments were performed in exactly the same way as described above for alkyne-labelled FAs, with the exception that heavy-isotope-labelled FAs were used instead of the FA;Ys. Lipid extracts were analysed directly by MS1/MS2 without click reaction and without multiplexing. For all isotope-labelled TGs, mfql search algorithms were written which identify the TGs by a combination of the unfragmented mass of the ammonium adducts in MS1 and combined neutral losses of the isotope-labelled FAs plus ammonia in MS2. These MS2 peaks were also used for quantification, relative to the corresponding MS2 fragment of the internal standard TG 50:1[D4]. For FA combination 11:0[D3]/16:0[13C16]/18:2[13C18], the search included single- and double-labelled species of 40–56 C-atoms, and for FA 16:0[13C16]/18:2[13C18]/19:1[D8], species of 48–57 C-atoms. Triple-labelled species were identified using individual targeted search files.

### Reporting summary

Further information on research design is available in the Nature Portfolio Reporting Summary linked to this article.

## Supplementary information

Supplementary Information Supplementary Tables 1–3, Spectrum 1 and Methods.

Reporting Summary

Supplementary Data 1 Processed and annotated mass spectrometric data file containing the source data for Fig. 3 and parts of Figs. 4, 5 and 6.

## Data Availability

Primary MS data .raw files and the LipidXplorer import .sc files and Excel calculation sheets are available from the publication date of the article in a publicly accessible archive: 10.22000/925. The annotated LipidXplorer output file that contains all primary identifications, raw intensities and pmol calculations of the time-resolved experiment that gives rise to Figs. 3, 4a–f and 6a,c,e is submitted as Supplementary Data 1.

## Code availability

LipidXplorer .mfql search files that contain the search algorithms used in this publication are available from the publication date of the article in a publicly accessible archive: 10.22000/925.
