# Supplementary material for: Triglyceride cycling enables modification of stored fatty acids
Source: Nat Metab. 2023 Apr 3;5(4):699–709. doi: 10.1038/s42255-023-00769-z (PMC10132980; doi:10.1038/s42255-023-00769-z)
Supplement: Supplementary file 1 — Supplementary Tables 1–3, Spectrum 1 and Methods. [file 42255_2023_769_MOESM1_ESM.pdf]

nature metabolism

Article https://doi.org/10.1038/s42255-023-00769-z

Triglyceride cycling enables modification of stored fatty acids

In the format provided by the authors and unedited

Supplementary materials:

a

		Label	0h	2h	4h	8h	24h
**alkyne**	**DB**	11:0;Y	1.32 ± 0.02	1.30 ± 0.02	1.31 ± 0.01	1.30 ± 0.01	1.46 ± 0.01
		16:0;Y	1.40 ± 0.01	1.42 ± 0.01	1.43 ± 0.01	1.43 ± 0.01	1.44 ± 0.01
		18:2;Y	3.19 ± 0.01	3.22 ± 0.01	3.25 ± 0.01	3.25 ± 0.01	3.26 ± 0.01
		all ;Y	1.80 ± 0.02	1.96 ± 0.02	2.06 ± 0.03	2.12 ± 0.01	2.41 ± 0.01
	**C-atoms**	11:0;Y	43.60 ± 0.01	43.70 ± 0.02	43.76 ± 0.03	43.82 ± 0.01	44.23 ± 0.06
		16:0;Y	49.10 ± 0.01	49.10 ± 0.01	49.13 ± 0.01	49.14 ± 0.01	49.21 ± 0.01
		18:2;Y	50.70 ± 0.01	50.72 ± 0.01	50.76 ± 0.02	50.76 ± 0.02	50.77 ± 0.02
		all ;Y	46.90 ± 0.04	47.87 ± 0.07	48.34 ± 0.04	48.66 ± 0.04	49.57 ± 0.02
			0h	2h	4h	8h	24h
**isotope**	**DB**	11:0	1.37 ± 0.02	1.37 ± 0.02	1.35 ± 0.01	1.31 ± 0.02	1.36 ± 0.03
		16:0	1.48 ± 0.02	1.46 ± 0.01	1.46 ± 0.01	1.47 ± 0.02	1.46 ± 0.03
		18:2	3.18 ± 0.02	3.19 ± 0.03	3.23 ± 0.02	3.23 ± 0.00	3.19 ± 0.03
		all iso	1.78 ± 0.02	1.86 ± 0.03	1.93 ± 0.01	1.96 ± 0.01	2.13 ± 0.05
	**C-atoms**	11:0	43.63 ± 0.03	43.69 ± 0.03	43.67 ± 0.03	43.65 ± 0.02	43.64 ± 0.01
		16:0	49.07 ± 0.04	49.06 ± 0.05	49.10 ± 0.03	49.12 ± 0.02	49.11 ± 0.02
		18:2	50.79 ± 0.07	50.81 ± 0.02	50.84 ± 0.03	50.90 ± 0.06	50.88 ± 0.03
		all iso	46.67 ± 0.05	47.18 ± 0.11	47.48 ± 0.05	47.79 ± 0.04	48.73 ± 0.06
**endogen.**	**DB**	all endo.	1.94 ± 0.01	1.99 ± 0.03	2.05 ± 0.01	2.14 ± 0.01	2.25 ± 0.04
	**C-atoms**	all endo.	49.12 ± 0.11	49.35 ± 0.12	49.48 ± 0.06	49.63 ± 0.05	50.03 ± 0.12

b

		Label	0h	2h	4h	8h	24h
**alkyne**	**DB**	16:0;Y	1.75 ± 0.02	1.72 ± 0.02	1.74 ± 0.03	1.76 ± 0.03	1.80 ± 0.00
		18:2;Y	3.37 ± 0.02	3.39 ± 0.03	3.37 ± 0.05	3.40 ± 0.03	3.50 ± 0.03
		19:1;Y	2.52 ± 0.03	2.53 ± 0.04	2.58 ± 0.09	2.57 ± 0.06	2.64 ± 0.02
		all ;Y	2.63 ± 0.03	2.64 ± 0.03	2.69 ± 0.05	2.70 ± 0.05	2.81 ± 0.01
	**C-atoms**	16:0;Y	49.16 ± 0.02	49.08 ± 0.04	49.11 ± 0.05	49.20 ± 0.1	49.24 ± 0.04
		18:2;Y	51.26 ± 0.03	51.15 ± 0.04	51.12 ± 0.07	51.25 ± 0.1	51.45 ± 0.04
		19:1;Y	51.75 ± 0.03	51.63 ± 0.05	51.74 ± 0.09	51.72 ± 0.12	51.80 ± 0.04
		all ;Y	50.78 ± 0.03	50.66 ± 0.04	50.70 ± 0.06	50.80 ± 0.11	50.99 ± 0.04
			0h	2h	4h	8h	24h
**isotope**	**DB**	16:0	1.55 ± 0.03	1.51 ± 0.04	1.51 ± 0.03	1.52 ± 0.02	1.47 ± 0.02
		18:2	3.29 ± 0.06	3.21 ± 0.06	3.24 ± 0.06	3.25 ± 0.04	3.33 ± 0.05
		19:1	2.37 ± 0.05	2.26 ± 0.02	2.29 ± 0.03	2.35 ± 0.07	2.43 ± 0.03
		all iso	2.35 ± 0.03	2.26 ± 0.03	2.29 ± 0.03	2.36 ± 0.05	2.45 ± 0.02
	**C-atoms**	16:0	49.25 ± 0.14	48.99 ± 0.1	49.13 ± 0.09	49.15 ± 0.08	49.19 ± 0.07
		18:2	50.93 ± 0.14	50.79 ± 0.12	50.80 ± 0.06	50.87 ± 0.13	51.02 ± 0.04
		19:1	52.30 ± 0.10	52.03 ± 0.13	52.17 ± 0.05	52.31 ± 0.07	52.36 ± 0.08
		all iso	51.36 ± 0.13	51.1 ± 0.12	51.27 ± 0.09	51.47 ± 0.09	51.61 ± 0.07
**endogen.**	**DB**	all endo.	2.44 ± 0.05	2.36 ± 0.09	2.37 ± 0.08	2.46 ± 0.06	2.60 ± 0.05
	**C-atoms**	all endo.	49.57 ± 0.18	49.53 ± 0.19	49.55 ± 0.1	49.61 ± 0.13	49.96 ± 0.11

Supplementary table 1: Comparison of fatty acid chain length and desaturation in labeled and unlabeled TG for FA combinations 11:0/16:0/18:2 (**a**) and 16:0/18:2/19:1 (**b**). 3T3-L1 cells were labeled for 1 h with alkyne-FA (alkyne) or heavy-isotope (isotope) labeled FA as indicated and chased for indicated periods between 0 h and 24 h. TGs were identified and quantified, and for each condition the weighted average number of C-atoms and double bonds (DB) were calculated. Numbers were calculated separately for TG containing either of the three labeled FA as indicated (Label) and for the sum of the labeled species (all ;Y, all iso). In the same way, the corresponding numbers for the unlabeled endogenous TG pool (endogen.) were calculated. The difference in numbers for endogenous lipids between part **a** and **b** is caused by the fact that these experiments were performed in separate differentiation batches of 3T3-L1 cells. All data are avg. +/- SD, n=12.

		Distribution of each species over the four chase times<br>% of species				Effect strength = deviation of species distribution from behavior of total pool<br>log2(%species/%total)				p-Value of distribution<br>-log 10 of p-values of species relative to total pool				Correlation to modified FA;Y<br>intensity ranking for FA			Effect strength x p-Value<br>ABS(log10(p-value) * log2(%species/%total))			
Species	mass (Da)	0h	6h	24h	48h	0h	6h	24h	48h	0h	6h	24h	48h	18:2;Y	20:3;Y	20:4;Y	0h	6h	24h	48h
TG 48:4;Y	969.8293	13	22	33	33	-0.03	-0.06	0.08	-0.03	0.80	1.55	1.86	0.59				0.02	0.10	0.16	0.02
TG 48:3;Y	971.8447	13	24	30	33	0.03	0.08	-0.05	-0.03	0.83	3.46	1.35	0.83	6			0.03	0.29	0.07	0.02
TG 48:2;Y	973.8609	14	24	29	34	0.08	0.07	-0.11	0.02	3.27	2.34	2.93	0.19				0.25	0.16	0.31	0.00
TG 49:4;Y	983.8463	17	22	32	29	0.37	-0.03	0.05	-0.21	5.09	0.49	0.44	1.97				1.89	0.02	0.02	0.41
TG 49:3;Y	985.8604	13	22	32	33	-0.02	-0.01	0.05	-0.03	0.39	0.22	1.31	0.33	7			0.01	0.00	0.07	0.01
TG 50:5;Y	995.8439	6	17	35	41	-1.03	-0.38	0.19	0.29	10.05	7.51	3.22	2.69			5	10.31	2.82	0.63	0.77
TG 50:4;Y	997.8593	14	23	32	31	0.03	0.06	0.07	-0.13	0.64	2.28	1.42	3.09	2			0.02	0.14	0.10	0.39
TG 50:3;Y	999.8749	15	23	31	31	0.14	0.06	0.03	-0.13	5.77	0.96	0.68	2.83	1			0.80	0.05	0.02	0.36
TG 50:2;Y	1001.889	14	26	27	32	0.11	0.22	-0.17	-0.05	2.52	3.08	4.25	0.31	5			0.27	0.68	0.74	0.02
TG 51:4;Y	1011.878	13	19	33	35	0.03	-0.22	0.08	0.05	0.33	6.08	0.87	0.44		7		0.01	1.32	0.07	0.02
TG 51:3;Y	1013.892	14	21	31	34	0.08	-0.07	-0.01	0.02	2.31	2.88	0.09	0.57	8			0.17	0.20	0.00	0.01
TG 52:8;Y	1017.829	21	27	29	23	0.68	0.25	-0.08	-0.56	1.30	0.60	0.22	1.34				0.89	0.15	0.02	0.75
TG 52:7;Y	1019.843	29	22	25	24	1.15	-0.03	-0.30	-0.49	6.41	0.17	2.73	4.08				7.35	0.01	0.83	1.99
➡ TG 52:6;Y	1021.86	9	13	30	48	-0.62	-0.80	-0.03	0.53	6.84	11.19	0.17	2.98			2	4.22	8.90	0.01	1.58
TG 52:5;Y	1023.875	6	15	36	43	-1.10	-0.58	0.21	0.36	9.56	12.14	2.98	3.79		1	1	10.52	7.00	0.63	1.37
⇨ TG 52:4;Y	1025.891	13	22	32	34	-0.05	-0.04	0.04	0.01	1.23	3.06	0.87	0.37	4	2		0.06	0.14	0.03	0.00
TG 52:3;Y	1027.904	14	24	30	33	0.08	0.07	-0.06	-0.03	1.54	1.63	0.82	0.61	3			0.12	0.12	0.05	0.02
TG 53:8;Y	1031.842	16	27	30	28	0.28	0.24	-0.05	-0.27	1.54	1.81	0.19	1.29				0.43	0.44	0.01	0.35
TG 53:7;Y	1033.858	19	26	29	26	0.50	0.22	-0.07	-0.38	4.25	2.54	0.28	2.57				2.13	0.57	0.02	0.98
TG 53:6;Y	1035.875	13	17	32	39	-0.08	-0.37	0.03	0.20	1.40	4.30	0.17	1.23				0.11	1.61	0.01	0.25
TG 53:5;Y	1037.89	8	16	34	42	-0.69	-0.46	0.14	0.31	8.85	7.16	2.11	3.53		5	7	6.13	3.29	0.30	1.09
TG 53:4;Y	1039.906	12	20	31	37	-0.13	-0.14	0.01	0.12	1.79	2.95	0.08	2.52		6		0.24	0.41	0.00	0.31
TG 53:3;Y	1041.922	11	19	29	41	-0.22	-0.23	-0.09	0.27	3.64	4.68	1.07	4.38				0.81	1.08	0.09	1.20
TG 54:9;Y	1043.844	11	20	33	36	-0.27	-0.16	0.09	0.11	0.25	0.34	0.23	0.25				0.07	0.05	0.02	0.03
TG 54:8;Y	1045.859	17	22	31	30	0.39	-0.04	0.01	-0.17	3.04	0.30	0.04	0.99				1.19	0.01	0.00	0.17
TG 54:7;Y	1047.874	19	22	31	29	0.51	-0.05	0.00	-0.22	4.78	0.53	0.00	2.17				2.43	0.03	0.00	0.48
TG 54:6;Y	1049.89	10	15	29	46	-0.39	-0.58	-0.10	0.46	5.90	9.40	0.76	3.43			4	2.28	5.41	0.07	1.57
TG 54:5;Y	1051.905	6	13	34	47	-1.17	-0.75	0.15	0.47	8.74	11.92	2.58	4.46		3	3	10.22	8.92	0.39	2.11
TG 54:4;Y	1053.921	12	20	30	37	-0.12	-0.15	-0.03	0.16	3.01	4.88	0.45	3.59		4		0.36	0.75	0.01	0.58
TG 54:3;Y	1055.936	11	20	26	43	-0.27	-0.17	-0.24	0.36	1.70	3.43	1.55	2.03				0.46	0.59	0.37	0.73
TG 55:8;Y	1059.875	13	22	33	32	0.03	-0.04	0.10	-0.08	0.13	0.20	0.47	0.49				0.00	0.01	0.05	0.04
TG 55:7;Y	1061.891	14	21	31	34	0.10	-0.07	-0.01	0.02	0.87	0.45	0.03	0.08				0.08	0.03	0.00	0.00
TG 55:6;Y	1063.906	11	17	29	43	-0.25	-0.40	-0.08	0.35	2.49	6.81	0.59	2.83				0.63	2.69	0.05	0.99
TG 55:5;Y	1065.922	8	15	34	43	-0.67	-0.59	0.13	0.36	8.09	7.81	1.52	3.52				5.41	4.60	0.20	1.26
TG 55:4;Y	1067.938	10	19	31	40	-0.35	-0.26	0.00	0.26	6.64	5.05	0.01	4.91				2.34	1.33	0.00	1.27
TG 55:3;Y	1069.955	8	16	30	46	-0.74	-0.49	-0.06	0.47	5.32	5.50	0.43	6.36				3.95	2.69	0.02	3.00
TG 56:9;Y	1071.874	13	24	33	30	-0.01	0.09	0.12	-0.18	0.10	0.64	1.07	1.41				0.00	0.06	0.13	0.25
TG 56:8;Y	1073.89	14	24	33	29	0.13	0.08	0.08	-0.20	3.12	1.59	0.56	1.28				0.41	0.13	0.04	0.25
TG 56:7;Y	1075.906	17	23	31	30	0.33	0.00	-0.01	-0.14	4.51	0.02	0.05	1.05				1.47	0.00	0.00	0.15
TG 56:6;Y	1077.922	10	14	29	47	-0.41	-0.71	-0.08	0.49	4.57	11.33	0.44	3.43				1.90	8.01	0.03	1.68
TG 56:5;Y	1079.938	4	12	34	50	-1.57	-0.95	0.14	0.57	8.57	10.46	2.30	4.90			6	13.43	9.93	0.33	2.82
TG 56:4;Y	1081.954	7	17	30	45	-0.94	-0.36	-0.03	0.44	7.53	6.19	0.34	4.88		8		7.08	2.25	0.01	2.14
TG 56:3;Y	1083.969	7	18	27	48	-0.88	-0.34	-0.17	0.51	6.34	4.93	2.00	4.03				5.57	1.66	0.34	2.05
TG 56:2;Y	1085.986	8	27	23	42	-0.69	0.29	-0.45	0.32	1.07	0.51	0.60	0.49				0.75	0.14	0.27	0.16
TG 58:10;Y	1097.891	3	15	37	44	-1.97	-0.55	0.26	0.40	4.77	2.26	1.69	1.97				9.40	1.25	0.45	0.79
TG 58:9;Y	1099.905	11	22	34	33	-0.27	-0.03	0.16	-0.04	2.77	0.30	1.23	0.19				0.74	0.01	0.20	0.01
TG 58:2;Y	1114.013	17	29	24	30	0.34	0.37	-0.34	-0.16	0.53	1.90	0.65	0.31				0.18	0.71	0.22	0.05
Total pool		13	22	31	34															

Supplementary table 2: This table refers to the data shown in Fig. 5. The left data block shows the distribution heat map of Fig. 5 with the underlying numbers, the second block the deviation of each species from the total, the third block the p-value of the second block data, and the fourth block shows the product of the second and third block. Between the third and fourth block are the intensity rankings for the appearance of the original label (FA 18:2;Y) and its metabolites FA 20:3;Y and 20:4;Y, as determined from spectra searching for those modified FA (see Extended Data Figures 8+9). N = 11-12, p-values are from unpaired, two-sided T-test.

Name	pmol/sample	*m/z* after click with C175-7x	charge state
CE 17:0;Y[D7]	26.0	817.806	+1
Cer 18:0;O2;Y/15:1[D8]	15.8	702.701	+1
DG 32:1;Y[D8]	14.6	745.695	+1
DG 38:2;Y2[13C3]	15.5	496.944	+2
LPC 19:1;Y[13C3]	37.4	355.268	+2
MG 19:1;Y[13C3]	27.1	544.473	+2
PA 32:1;Y[D8]	12.8	825.662	+1
PA 38:2;Y2[13C3]	27.6	536.927	+2
PC 32:1;Y[D8]	113.2	455.879	+2
PC 38:2;Y2[13C3]	49.4	386.314	+3
PE 32:1;Y[D8]	36.0	868.704	+1
PE 38:2;Y2[13C3]	26.1	558.448	+2
PI 32:1;Y[D8]	30.7	987.715	+1
PS 32:1;Y[D8]	33.9	912.694	+1
TG 48:1;Y[D8]	103.0	983.925	+1
TG 49:1;Y2[D8]	20.3	584.548	+2
TG 57:3;Y3[13C3]	32.7	481.101	+3
TG 49:1;Y3[D5]	35.3	445.743	+3
TG 50:1[D4]	1600	854.81 (xNH_4^+)	+1

Supplementary table 3: Composition of the internal standard mixture that was added to each sample.

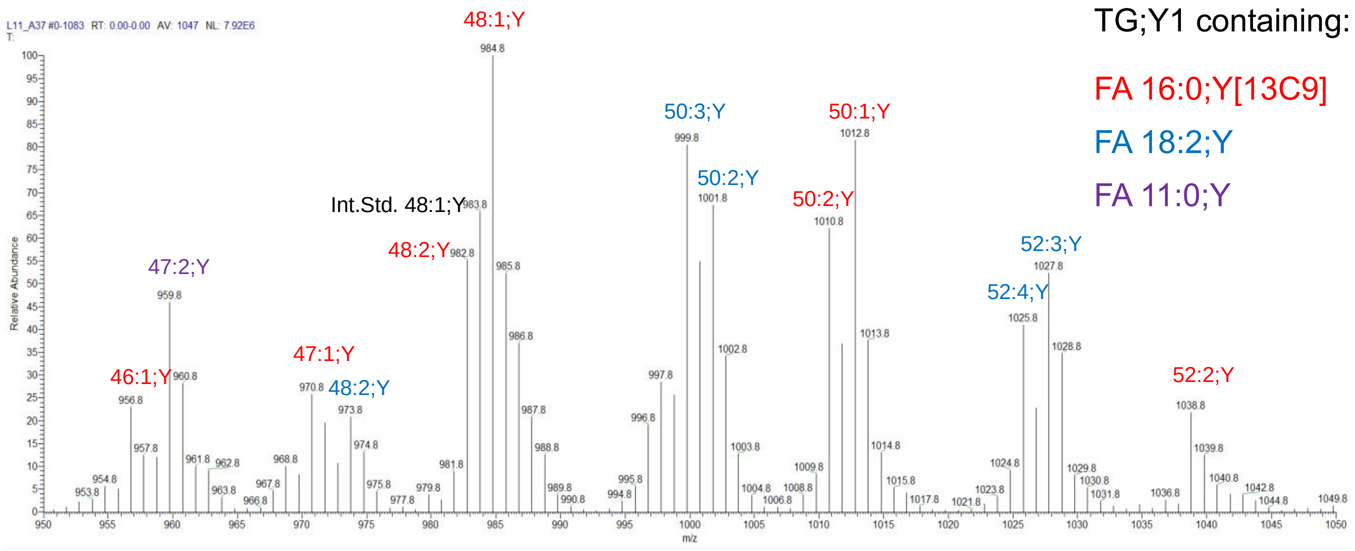

Supplementary spectrum 1, refers to Extended data Figure 6b: Identification of labeled TG;Y1 in alkyne labeling experiments. 3T3-L1 cells were labeled with alkyne FA (11:0;Y, 16:0;Y, 18:2;Y, each at 50 µM) for 1h, and lipid extracts were clicked with C175 reagents. MS2 spectra were recorded and, using the ion map function of the Themo Xcalibur software, fragmentation spectra were reconstructed. The figure shows the m/z 950-1050 region of the neutral loss m/z 73.1 spectrum containing the alkyne-labeled neutral lipids clicked to C175-73. The annotation shows the most prominent peaks and indicates the underlying species and the labeled fatty acid that they contain.

Supplementary methods 1: Details of mfql writing for alkyne- and isotope-labeled lipid species.

The following example identifies, in a multiplex sample using the C175-7x reagent series, double-labeled double-charged TGs that contain 9 x ^{13}C, 43 total C and a total of 0-3 double bonds in the fatty acids:

```
QUERYNAME = a11a16Y2TAG43;
DEFINE PR1 = 'C[53] Ci9 H[96..110] D8 N[8] O[6]' WITH DBR = (8,10) , CHG = 2
#Comment: PR1 is the double-clicked un-fragmented TG. The 9 x 13C limit the detection to the species labeled with FA 16:0;Y[13C9]
DEFINE NL1 = 'C[45] Ci9 H[74..88] D8 N6 O6'  WITH DBR = (10,12) ,  CHG = 2;
DEFINE NL2 = 'C[45] Ci9 H[78..92] D4 N6 O6'  WITH DBR = (10,12) , CHG = 2;
DEFINE NL3 = 'C[45] Ci9 H[80..94] D2 N6 O6'  WITH DBR = (10,12) , CHG = 2;
DEFINE NL4 = 'C[45] Ci9 H[82..96] N6 O6'  WITH DBR = (10,12) , CHG = 2;
#Comment: These are the four multiplexed fragments after NL of two dimethylethylamines from the C175 reagents.

IDENTIFY
PR1 IN MS1+
AND NL1 IN MS2+
AND NL2 IN MS2+
AND NL3 IN MS2+
AND NL4 IN MS2+
#Comment: This is the actual search for the precursor and the fragments in MS1 and MS2, respectively.

SUCHTHAT
isEven(PR1.chemsc[H]) AND
NL1.chemsc + 'C8 H22 N2' == PR1.chemsc AND
NL2.chemsc + 'C8 H18 D4 N2' == PR1.chemsc AND
NL3.chemsc + 'C8 H16 D6 N2' == PR1.chemsc AND
NL4.chemsc + 'C8 H14 D8 N2' == PR1.chemsc
#Comment: This section makes sure that precurosor peaks and fragments belong to each other.

REPORT
NAME = "TG %d:%d;Y2" % ((PR1.chemsc[C] - 10), (PR1.chemsc[db] - 8));
ALTNAME = "dlTG %d:%d" % ((PR1.chemsc[C] - 10), (PR1.chemsc[db] - 4));
CLASS = "TAGY2";
SAMPLE = "1";
IS = "0";
chemsc = PR1.chemsc;
C = "%d" %  (PR1.chemsc[C] - 10);
db = "%d" %  (PR1.chemsc[db] - 8);
tb = 2;
FA110Y = "1";
FA160Y = "1";
FA182Y = "0";
MASS = "%4.4f" % "(PR1.mass)";
ERROR = "%2.2f ppm" % "(PR1.errppm)";
ISpmol = 20.34;
#Comment: This part of the report section gives names and basic parameters of the TGs, and some logical operators for further calculations.

INT73 = NL1.intensity;
INT75 = NL2.intensity;
INT76 = NL3.intensity;
INT77 = NL4.intensity; ;
#Comment: this part reports the four intensities of the four multiplex channels.
```

Search for heavy isotope-labeled species: Below is the example of single-labeled TG containing the FA 16:0[13C16]. Note that ^{13}C is denoted as “Ci”.

```
QUERYNAME = TAG13C16NL;
DEFINE PR1 = 'C[33..43] Ci16 H[80..120]  N[1] O[6]' WITH DBR = (1.5,7.5), CHG = 1; #ammonia adducts of TG with 16x13C
DEFINE NL = 'Ci16 H[35]  N[1] O[2]' WITH CHG = 0; #comment: this is the key identifier, i.e. the neutral loss of labeled FA together with ammonia.

IDENTIFY
PR1 IN MS1+
AND NL in MS2+

SUCHTHAT
isOdd(PR1.chemsc[C]) AND
isEven(PR1.chemsc[H])

REPORT
NAME = "TG %d:%d" % ((PR1.chemsc[C] + 13), (PR1.chemsc[db] - 1.5));
CLASS = "TACG";
SAMPLE = "1";
IS = "0";
chemsc = PR1.chemsc;
C = "%d" %  (PR1.chemsc[C] + 13);
db = "%d" %  (PR1.chemsc[db] - 1);
FA110 = "0";
FA160 = "1";
FA182 = "0";
MASS = "%4.4f" % "(PR1.mass)";
ERROR = "%2.2f ppm" % "(PR1.errppm)";
ISpmol = 1600;
INT = NL.intensity; ;
```
