# Supplementary material for: Triglyceride cycling enables modification of stored fatty acids
Source: Nat Metab. 2023 Apr 3;5(4):699–709. doi: 10.1038/s42255-023-00769-z (PMC10132980; doi:10.1038/s42255-023-00769-z)
Supplement: Supplementary file 2 — Reporting Summary [file 42255_2023_769_MOESM2_ESM.pdf]

nature portfolio

Corresponding author(s): Christoph Thiele

Last updated by author(s): Feb 23, 2023

Reporting Summary

Nature Portfolio wishes to improve the reproducibility of the work that we publish. This form provides structure for consistency and transparency in reporting. For further information on Nature Portfolio policies, see our Editorial Policies and the Editorial Policy Checklist.

Statistics

For all statistical analyses, confirm that the following items are present in the figure legend, table legend, main text, or Methods section.

n/a	Confirmed	
☐	☒	The exact sample size (*n*) for each experimental group/condition, given as a discrete number and unit of measurement
☐	☒	A statement on whether measurements were taken from distinct samples or whether the same sample was measured repeatedly
☒	☐	The statistical test(s) used AND whether they are one- or two-sided<br>*Only common tests should be described solely by name; describe more complex techniques in the Methods section.*
☒	☐	A description of all covariates tested
☒	☐	A description of any assumptions or corrections, such as tests of normality and adjustment for multiple comparisons
☐	☒	A full description of the statistical parameters including central tendency (e.g. means) or other basic estimates (e.g. regression coefficient) AND variation (e.g. standard deviation) or associated estimates of uncertainty (e.g. confidence intervals)
☒	☐	For null hypothesis testing, the test statistic (e.g. *F*, *t*, *r*) with confidence intervals, effect sizes, degrees of freedom and *P* value noted<br>*Give P values as exact values whenever suitable.*
☒	☐	For Bayesian analysis, information on the choice of priors and Markov chain Monte Carlo settings
☒	☐	For hierarchical and complex designs, identification of the appropriate level for tests and full reporting of outcomes
☒	☐	Estimates of effect sizes (e.g. Cohen's *d*, Pearson's *r*), indicating how they were calculated

Our web collection on statistics for biologists contains articles on many of the points above.

Software and code

Policy information about availability of computer code

Data collection	Thermo Q Exactive Plus software version 2.8 SP1 Built 2806
Data analysis	LipidXplorer 1.2.7, MS Excel 2016, Thermo Xcalibur 4.0. Code: LipidXplorer .mfql search files that contain the search algorithms used in this publication are available from the publication date of the article in a publicly accessible archive (DOI) 10.22000/925.

For manuscripts utilizing custom algorithms or software that are central to the research but not yet described in published literature, software must be made available to editors and reviewers. We strongly encourage code deposition in a community repository (e.g. GitHub). See the Nature Portfolio guidelines for submitting code & software for further information.

Data

Policy information about availability of data

All manuscripts must include a data availability statement. This statement should provide the following information, where applicable:

- Accession codes, unique identifiers, or web links for publicly available datasets
- A description of any restrictions on data availability
- For clinical datasets or third party data, please ensure that the statement adheres to our policy

Data: Primary MS data .raw files and the LipidXplorer import .sc files and Excel calculation sheets are available from the publication date of the article in a publicly accessible archive (DOI) 10.22000/925.

Human research participants

Policy information about studies involving human research participants and Sex and Gender in Research.

Reporting on sex and gender	*Use the terms sex (biological attribute) and gender (shaped by social and cultural circumstances) carefully in order to avoid confusing both terms. Indicate if findings apply to only one sex or gender; describe whether sex and gender were considered in study design whether sex and/or gender was determined based on self-reporting or assigned and methods used. Provide in the source data disaggregated sex and gender data where this information has been collected, and consent has been obtained for sharing of individual-level data; provide overall numbers in this Reporting Summary. Please state if this information has not been collected. Report sex- and gender-based analyses where performed, justify reasons for lack of sex- and gender-based analysis.*
Population characteristics	*Describe the covariate-relevant population characteristics of the human research participants (e.g. age, genotypic information, past and current diagnosis and treatment categories). If you filled out the behavioural & social sciences study design questions and have nothing to add here, write "See above."*
Recruitment	*Describe how participants were recruited. Outline any potential self-selection bias or other biases that may be present and how these are likely to impact results.*
Ethics oversight	*Identify the organization(s) that approved the study protocol.*

Note that full information on the approval of the study protocol must also be provided in the manuscript.

Field-specific reporting

Please select the one below that is the best fit for your research. If you are not sure, read the appropriate sections before making your selection.

☒ Life sciences ☐ Behavioural & social sciences ☐ Ecological, evolutionary & environmental sciences

For a reference copy of the document with all sections, see nature.com/documents/nr-reporting-summary-flat.pdf

Life sciences study design

All studies must disclose on these points even when the disclosure is negative.

Sample size	To estimate the necessary sample size, we assumed a difference in experimental values between two experimental groups of 20% and a SD of 10% for each group. With these asssumptions, a power analysis (using G*power 3.1.9.7) indicates a necessary sample size of 4 to achieve a p value of 0.95. Most data in the publication have a sample size of n=11-12, others (Fig. 2b,c and Fig 4 g-k) are at n=4. Actual effect sizes are much larger than the assumed 20% difference, indicating that n=4 is sufficient. The key data in this publication, i.e. the TG cycling data in Fig. 4 and the fatty acid modification data in Fig. 6 are normalized to total labeled TG or total labeled FA, which leads to very samll residual variations. Resulting SD-values are very small, as can be seen in the Figures 4 and 6.
Data exclusions	The import function in LipidXplorer uses a global thresholding that excludes peaks smaller than 1000 cps. In Fig. 4 and 6, when the legend indicates n = 11-12, two wells from the 48-well plate were excluded because they contained less than 50% of the average amount of labeled material. This is usually due to cell loss in wash steps. Otherwise no data were excluded.
Replication	The calibration experiment was repeated in different 3T3-L1 adipocytes and in freshly isolated hepatocytes , each in two different combinations of labeled fatty acids. All experiments worked well and allowed clear assignment of identified lipid species to the individual labeled fatty acids. The time resolved experiments were done with at least three exact repetitions. All repetitions fully support the presented data.
Randomization	No randomization. In these cell culture experiments, the only possible randomization would be the distribution of different chase times over the positions of the multiwell plates. We did not do that because the risk of handling mistakes would be too large.
Blinding	No blinding. The experimentator cannot perform the cell culture pulse-chase experiment if they do not know what the samples contain. All data were analyzed by a person different from the experimentator.

Reporting for specific materials, systems and methods

We require information from authors about some types of materials, experimental systems and methods used in many studies. Here, indicate whether each material, system or method listed is relevant to your study. If you are not sure if a list item applies to your research, read the appropriate section before selecting a response.

Materials & experimental systems

n/a	Involved in the study
☒	☐ Antibodies
☐	☒ Eukaryotic cell lines
☒	☐ Palaeontology and archaeology
☐	☒ Animals and other organisms
☒	☐ Clinical data
☒	☐ Dual use research of concern

Methods

n/a	Involved in the study
☒	☐ ChIP-seq
☒	☐ Flow cytometry
☒	☐ MRI-based neuroimaging

Eukaryotic cell lines

Policy information about cell lines and Sex and Gender in Research

Cell line source(s)	3T3-L1, originally bought from ATCC, subcloned and further propagated.
Authentication	Cells are tested for the ability to differentiate into an adipocyte phenotype upon induction with insulin, dexamethason and IBMX. Mouse species origin was checked by mRNA sequencing.
Mycoplasma contamination	Routine tests for mycoplasma are performed by PCR and were all negative
Commonly misidentified lines (See ICLAC register)	none

Animals and other research organisms

Policy information about studies involving animals; ARRIVE guidelines recommended for reporting animal research, and Sex and Gender in Research

Laboratory animals	Mice were maintained and bred in the LIMES GRC animal facility. The mice had free access to standard rodent diets and water. Animals were housed in a 12:12 light-dark (LD) cycle, at 23 ± 1°C. All animal studies were performed according to German animal welfare laws. Animal experiments were permitted by the Landesamt für Natur, Umwelt und Verbraucherschutz (LANUV) Nordrhein-Westfalen, Germany, 81-02.04.2021.A166.
Wild animals	not applicable
Reporting on sex	This study used mice to collect white and brown adipose tissue for generation of white or brown adipocytes for cell culture experiments. Male mice were used to minimize cycle effects on metabolic behavior of cells. We are not aware of sex-related limitations of the applicability of results obtained from such primary culture cells.
Field-collected samples	not applicable
Ethics oversight	All animal studies were performed according to German animal welfare laws. Animal experiments were permitted by the Landesamt für Natur, Umwelt und Verbraucherschutz (LANUV) Nordrhein-Westfalen, Germany, 81-02.04.2021.A166. Tissue collection experiments do not require additional ethics oversight.

Note that full information on the approval of the study protocol must also be provided in the manuscript.
